# Structural and biochemical analysis reveals how ferulic acid improves catalytic efficiency of *Humicola* *grisea* xylanase

**DOI:** 10.1038/s41598-022-15175-w

**Published:** 2022-07-06

**Authors:** Izadora Cristina Moreira Oliveira, Aisel Valle Garay, Amanda Araújo Souza, Napoleão Fonseca Valadares, João Alexandre Ribeiro Gonçalves Barbosa, Fabrícia Paula Faria, Sonia Maria Freitas

**Affiliations:** 1 Department of Cell Biology, Laboratory of Biophysics, Biology Institute, University of Brasília (UnB), Quadra 604, Asa Norte, Bloco J 1° andar, Brasília, DF 70910-900 Brazil; 2 Brazilian Biosciences National Laboratory (LNBio), National Center for Research in Energy and Materials (CNPEM), Campinas, SP 13083-970 Brazil; 3 Department of Biochemistry and Molecular Biology, Biological Sciences Institute, Federal University of Goiás, Goiânia, Goiás 74690-900 Brazil

**Keywords:** Enzymes, Computational biophysics, Molecular biophysics, Protein structure predictions, Biophysical methods, Isolation, separation and purification, Biochemistry, Biophysics, Biotechnology

## Abstract

*Humicola* *grisea* var. *thermoidea* is an aerobic and thermophilic fungus that secretes the GH11 xylanase HXYN2 in the presence of sugarcane bagasse. In this study, HXYN2 was expressed in *Pichia* *pastoris* and characterized biochemically and structurally in the presence of beechwood xylan substrate and ferulic acid (FA). HXYN2 is a thermally stable protein, as indicated by circular dichroism, with greater activity in the range of 40–50 °C and pH 5.0–9.0, with optimal temperature and pH of 50 °C and 6.0, respectively. FA resulted in a 75% increase in enzyme activity and a 2.5-fold increase in catalytic velocity, catalytic efficiency, and catalytic rate constant (k_cat_), with no alteration in enzyme affinity for the substrate. Fluorescence quenching indicated that FA forms a complex with HXYN2 interacting with solvent-exposed tryptophan residues. The binding constants ranged from moderate (pH 7.0 and 9.0) to strong (pH 4.0) affinity. Isothermal titration calorimetry, structural models and molecular docking suggested that hydrogen bonds and hydrophobic interactions occur in the aglycone region inducing conformational changes in the active site driven by initial and final enthalpy- and entropy processes, respectively. These results indicate a potential for biotechnological application for HXYN2, such as in the bioconversion of plant residues rich in ferulic acid.

## Introduction

Cellulose and hemicellulose residues from plant biomass are most commonly degraded in industrial applications by enzymes derived from filamentous fungi. Thermophilic fungi stand out as major producers of hemicellulases that exhibit high degradation efficiency at different pHs and temperatures^1^. Hemicellulose is a branched heteropolysaccharide that interacts with the lignocellulosic matrix through covalent interactions^2^. Xylan is the main hemicellulose present in plant cell walls with a varied structure consisting of a linear d-xylopyranose backbone linked by β-1,4 glycosidic bond. This backbone possesses branches in its 2- and/or 3-hydroxy groups, with side chain substituents, such as α-l-arabinofuranosyl, *O*-acetyl, α-1,2-linked glucuronic acid and 4-*O*-methyl-d-glucuronic acid groups^3^.

The principal enzymes for xylan degradation comprise endoxylanases (EC 3.2.1.8), which are produced by fungi and bacteria, cleaving internal β-1,4-xylosidic bonds in the main xylan chain, and β-xylosidases (EC 3.2.1.37), in turn cleaving β-1,4-xylo-oligosaccharides into xylose^4^. Endoxylanases have been classified according to the CAZy database into several families, including the GH10 and GH11^5,6^. Members of the GH11 family are characterized by high pI value (8.0–9.0), low molecular mass (≤ 30 kDa) and high β-sheet (~ 65%), and low α-helix content^7,8^. The three-dimensional structure of endo-1,4-beta-xylanases of the GH11 family consists of a single domain of β-jelly-roll structure, composed of two twisted anti-parallel β-sheets forming a long and deep cleft. The unique α-helix is packed under the β-sheet B^8–10^.

The active site of GH11 xylanases consists of an open cleft that extends along the length of the protein where four or more xylose residues can bind^11^. The substrate binding site is divided into contiguous binding subsites called (+ n), (+ 2), (+ 1) to (− 1), (− 2), (− n), where each xylose is anchored by non-covalent interactions^8^. Hydrolysis of the glycosidic bond occurs between (+ 1) and (−1) subsites^12,13^ by two catalytic Glu residues located at the reducing end or “aglycone region” (subsites + n, + 2, + 1) and at the non-reducing end or “glycone region” (subsites − n, − 2, − 1), respectively.

*Humicola* *grisea* *var.* *thermoidea* is an aerobic and thermophilic fungus with optimal growth at 40–42 °C. This strain was originally isolated from composting material^14^ and classified according to its ability to produce enzymes, mainly endoxylanases, at high temperatures^15,16^. When grown in sugarcane bagasse, this fungus secretes a GH11 xylanase known as HXYN2, with 227 amino acids, a molecular mass of 23 kDa, pI of 6.1, and optimal temperature and pH of 65 °C and 5.5, respectively^17^. The *H.* *grisea* xylanase 2 cDNA (*hxyn2*) was previously expressed in the fungus *Trichoderma* *reesei*, resulting in an active enzyme^18^. This gene was also expressed in *Pichia* *pastoris* resulting in a recombinant enzyme with a theoretical pI of 8.6, and optimal pH and temperature of 6.5 and 60 ºC, respectively^19,20^.

HXYN2 secreted by *P.* *pastoris* has been used in Organosolv pulp bio-bleaching processes for paper production^21^. In this study, HXYN2 showed greater efficiency in reducing the pulp viscosity in comparison with commercial xylanase, favoring the bleaching process. Sequential hydrolysis of beechwood and oat spelt, in which HXYN2 was added prior to β-xylosidase (HXYLA), also promoted a greater release of reducing sugar than when HXYN2 and HXYLA were employed separately and/or simultaneously^22^. Hydrolysis of steam explosion-pretreated sugarcane bagasse has also been shown to be promoted by an enzyme cocktail of recombinants HXYN2, HXYLA, and α-l-arabinofuranosidase (ABF3) from *Penicillium* *purpurogenum*. This hydrolysis resulted in 50% increase in glucose release, when added prior to commercial cellulases (Accellerase 1500, Dupont, Rochester, NY, USA) in sequential reactions. In this study, the glucose yield was 14.6% when all enzymes were used simultaneously^20,23^. Such results highlight the high biotechnological potential of HXYN2 when employed rationally in different process stages.

The objective of our study was to investigate the effect of ferulic acid (FA) on catalytic efficiency of *Humicola* *grisea* xylanase, based on physicochemical, biochemical and structural characteristics. These characteristics are shown to be important for biotechnological applications of HXYN2 in various industrial processes, principally in the bioconversion of plant residues rich in FA and optimization of biorefining processes through effective enzymatic treatment.

## Results

### Enzyme production and purification

**Figure 1:**
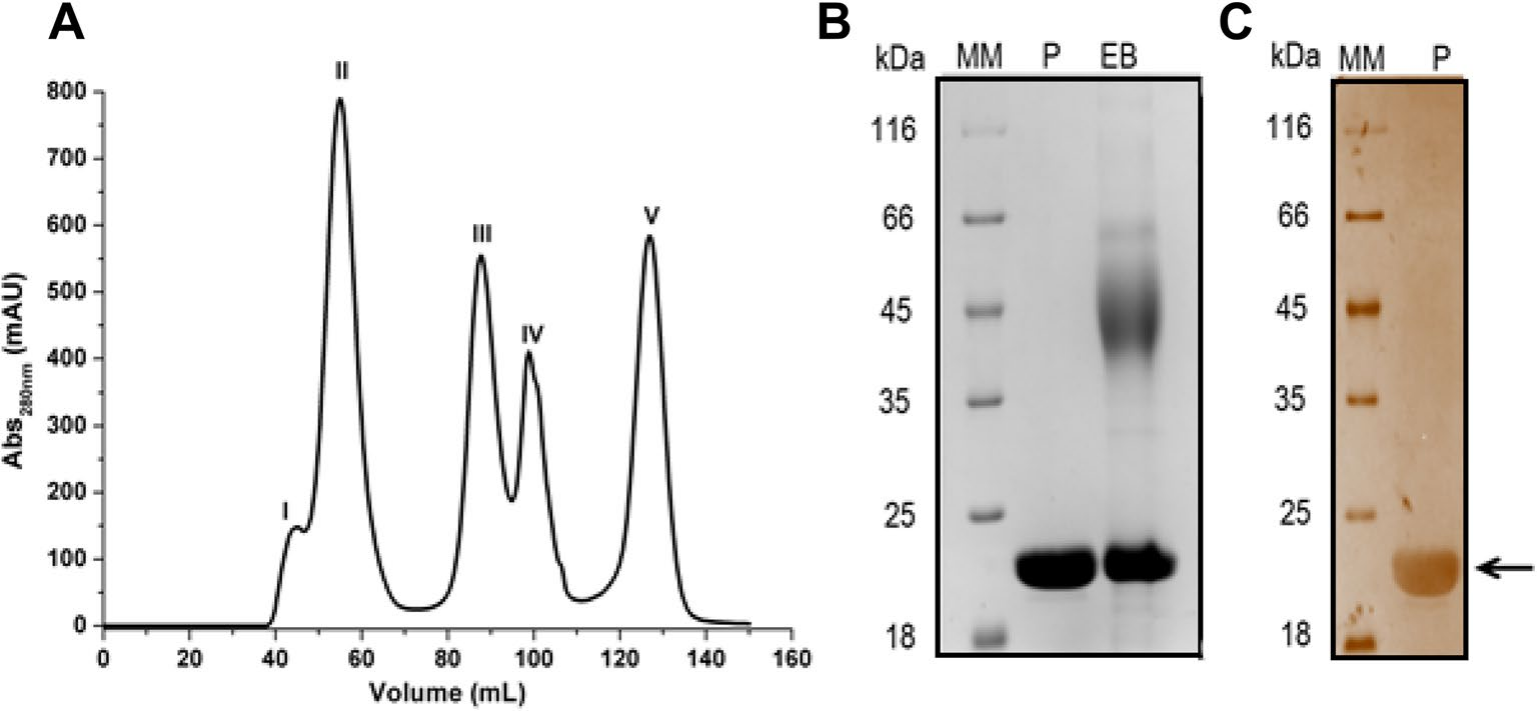
Purification and purity analysis of recombinant HXYN2. (**A**) Chromatographic profile of proteins eluted from a Superdex S75 Hiload 16/600 column. The fractions of peak V correspond to HXYN2. (**B**) Analysis of this sample in SDS-PAGE (12%) stained with Coomassie blue: crude extract produced by yeast (EB), purified HXYN2 and concentrated 5× (P), and molecular weight markers (MM). (**C**) SDS PAGE (12%) stained with silver nitrate: molecular weight markers (MM), purified HXYN2 and concentrated 5× (P). Gels were cropped from the original gels that retain P, EB, and MM bands, as shown in Supplementary Fig. S1.

Heterologous expression of HXYN2 was performed, with the culture supernatant showing an enzymatic activity of 354 U/mL. Purification of HXYN2 was accomplished in a single step by molecular exclusion using a Superdex S-75 column, with a 30% yield (Supplementary Table S1). The fractions corresponding to peak V (Fig. 1A) showed xylanase activity of 415 U/mL. Electrophoresis under denaturing conditions revealed a single band with an approximate molecular mass of 23 kDa (Fig. 1B,C).

The values of ε_280nm_ and 0.1% absorptivity at 280 nm of HXYN2 were calculated as 6,7623.60 L/mol.cm and 2.64 mL/mg.cm, respectively (Supplementary Fig. S2). These values were taken into account for determining the protein concentration in all the following assays.

### Molecular modeling of HXYN2 structure

**Figure 2:**
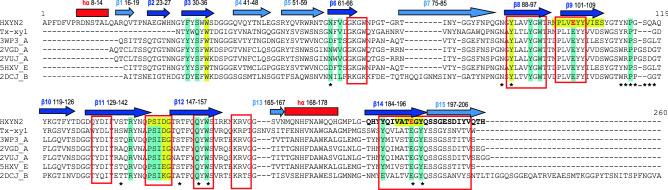
Multiple alignment of amino acid sequences of HXYN2 and xylanase templates. The amino acid sequences of the comparative xylanases were obtained from the non-redundant protein databases using the NCBI BLAST protein server, BLASTp (https://blast.ncbi.nlm.nih.gov/Blast.cgi), from the National Library of Medicine of the USA and were aligned with HXYN2 using the program MEGAv7^28^. Gaps are identified with a dash. Secondary structures are identified by a red bar (α-helix), navy blue arrows (six β-strands of A β-sheet), and blue arrows (nine β-strands of B β-sheet). The regions with highest sequence similarities are enclosed in a red square, the highly conserved sequence “PSIXG” highlighted in yellow, and the catalytic Glu residues in red. The amino acids marked with an asterisk are involved in FA recognition, with the exception of R148. Strictly conserved residues that participate in xylan binding are shaded in turquoise. Endo-1,4-β-xylanase template PDB structures were downloaded from https://www.rcsb.org/structure with identifiers: 3WP3_A of XylC from *Talaromyces* *cellulolyticus*; 2VGD_A of NpXyn11A from *Neocallimastix* *patriciarum*; 2VUJ_A of EvXyn11 environmental xylanase expressed in *Escherichia* *coli*; 5HXV_E of XylC from *Talaromyces* *cellulolyticus*; and 2DCJ_B of XynJ from *Bacillus* *sp.* *41* *M-1.*

The previously reported structural model of HXYN2^24^ has been updated based on current crystallographic structures of xylanases and the employment of new automated and accurate methodologies for predicting protein structures. This model was later used to obtain the xylanase-FA complex required to analyze and corroborate the experimental results presented here. Five structural HXYN2 models were built according to the templates shown in Fig. 2 and Supplementary Table S2.

**Figure 3:**
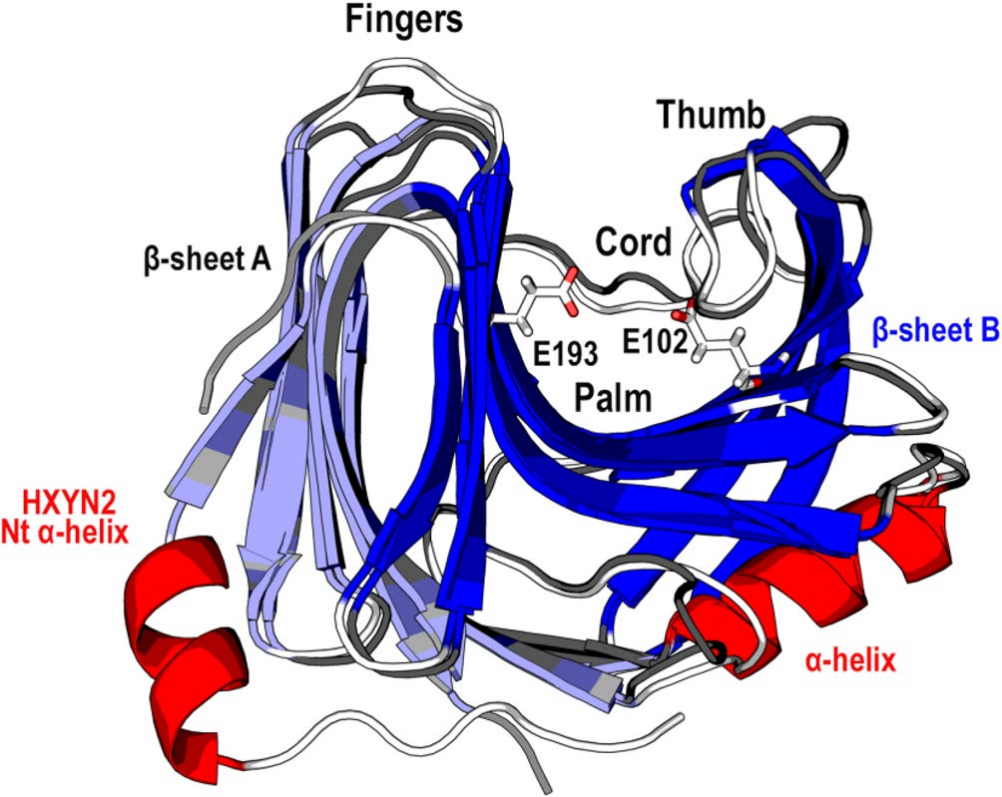
Three-dimensional model of HXYN2 (loops and β-turns in white) superimposed with its template (loops and β-turns in dark grey) from https://www.rcsb.org/structure with identifier PDB: 3WP3. The β-jelly-roll super-fold is formed by two anti-parallel β-sheets, namely A (in light blue) and B (in blue). The “fingers” form loops between β-sheets A and B, the “palm” represents the cleft, and a loop forms the “thumb”. An additional helix was predicted in the N-terminal region. The two catalytic Glu residues, E102 and E193, are represented by sticks. The figure was generated using *PyMol* *Molecular* *Graphics* *System* *version* *2.1.1* (http://www.pymol.org).

The GH11 xylanase from *Thermobacillus* *xylanilyticus* (Tx-xyl) was considered for residue numbering in the multiple sequence alignment (Fig. 2)^8,25^. The highest sequence similarity occurs in the β-strands that form the “palm” and deep cleft in the β-sheet B, such as: β8, β9, β11, β12, and β14-β15 at the C-terminal end. The “thumb” between the β11–β12 β-strands shows a highly conserved consensus sequence “PSIXG” among GH11 xylanases^8,26^. In contrast, the more variable regions include the loops that form the “fingers” and different external β-strands that form the β-sheet A (Figs. 2 and 3). The two catalytic Glu residues and highly conserved substrate-binding environment of the HXYN2 models^8,11,27^ are highlighted in red and enclosed in a red square, respectively.

**Figure 4:**
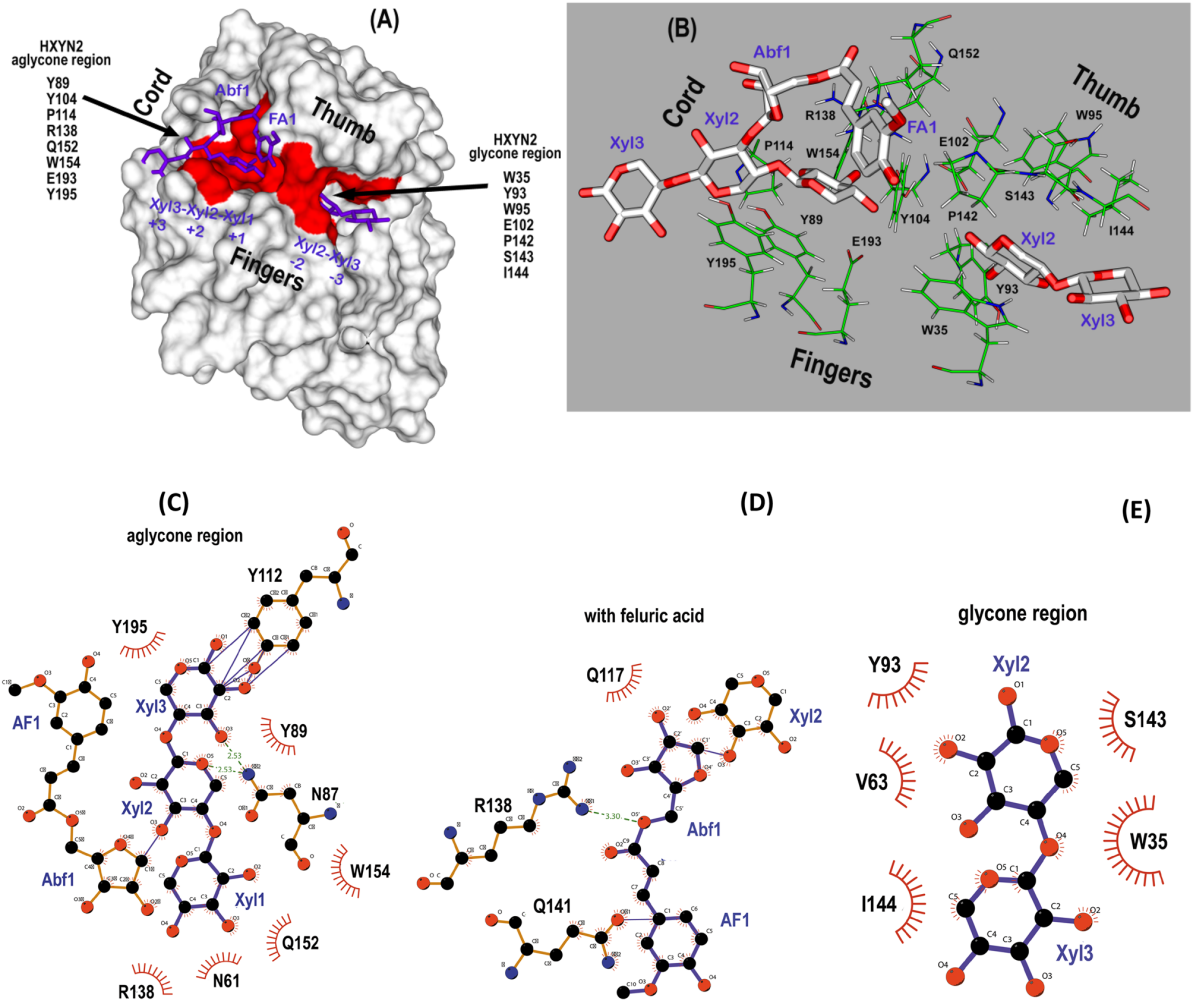
Interaction of the HXYN2 model with xylooligosaccharide substrate and FA from the NpXyn11A template (https://www.rcsb.org/strucutre) with identifier (PDB: 2VGD_A) (Supplementary Table S2). (**A**) The surface of HXYN2 is represented in white, with the strictly conserved residues of the aglycone and glycone regions represented in red. Ligands are represented in a purple stick format. The xylooligosaccharide (Xyl3–Xyl2–Xyl1), with xylose sugars 1 (Xyl1) at the reducing end sugar, is located in the aglycone region, with each monosaccharide located in its corresponding + 3, + 2, + 1 subsite. Arabinofuranose (Abf1) and ferulic acid (FA1) are also labeled at the aglycone site. Xylobiose (Xyl2–Xyl3), with the non-reducing end in Xyl3, is shown binding to − 2 and − 3 subsites of the glycone region. (**B**) The structural arrangement of conserved residues in the aglycone and glycone regions are shown in green and their interaction with ligands are represented as blue sticks. Figures (**A,B**) were generated using *PyMol* *Molecular* *Graphics* *System* *version* *2.1.1* (http://www.pymol.org). (**C**) Interactions between HXYN2 and Xyl3-Xyl2-Xyl1, (**D**) and Xyl2-Xyl3, (**E**) and Abf1 and FA1 are shown by LIGPLOT representations^31^. Amino acid residues of HXYN2 and ligands are labeled; the hydrogen bonding and the hydrophobic interactions are represented as dashed lines and a striped hemisphere, respectively.

The best score for the HXYN2 structural model (model 1) was achieved using the GH11 Xylanase XylC from mesophilic *Talaromyces* *cellulolyticus* as a template (PDB: 3WP3) (Supplementary Table S2)^29^. The substrate-binding site and catalytic residues of HXYN2, as well as those potentially involved in interactions with FA, were identified by superimposing HXYN2 model 2 with its NpXyn11A template (PDB: 2VGD_A), a complex structure with xylobiose and feruloyl-arabino-xylotriose (Fig. 4). The substrate interface analysis and identification of catalytic residues were performed by superimposition of the HXYN2 model with GH11 xylanase SoXyn11B from *Streptomyces* *olivaceoviridis* *E-86* in complexes with alpha-l-3-arabinofuranosyl xylotetraose (PDB: 7DFN) and with 4-*o*-methyl-alpha-d-3-glucuronopyranosyl xylotetraose (PDB: 7DFO) (Supplementary Fig. S3)^30^.

As with other GH11 xylanases, the residues of the “aglycone” (P114, R138, Q152, W154, E193, Y89, Y104, and Y195) and “glycone” (Y93, E102, P142, S143, I144, W39, and W95) sites are strictly conserved in the template and in the HXYN2 model (Fig. 4A). In the HXYN2 model, the catalytic residue E102 is deprotonated (catalytic nucleophile) and E193 is protonated (catalytic acid–base), located in the β-strands β9 and β14, respectively (Fig. 4B).

Xylose at the −  1 subsite of the template interacts with the E102 catalytic residue, with N61, V63, P142, and R138 residues superimposing with SoXyn11B enzyme–substrate complexes (Supplementary Fig. S3). At the – 2 subsite, Xyl2 interacts with W35, V63, Y93, S143 and I144 residues, while Xyl3 at subsite – 3 interacts only with I144 residue (Fig. 4B,D , and Supplementary Fig. S3). The interaction with xylotriose, branched with arabinofuranose and FA at C3 of xylose 2 are shown (Fig. 4B,C).

### Effect of concentration, pH, and temperature on HXYN2 activity and its thermal stability

**Figure 5:**
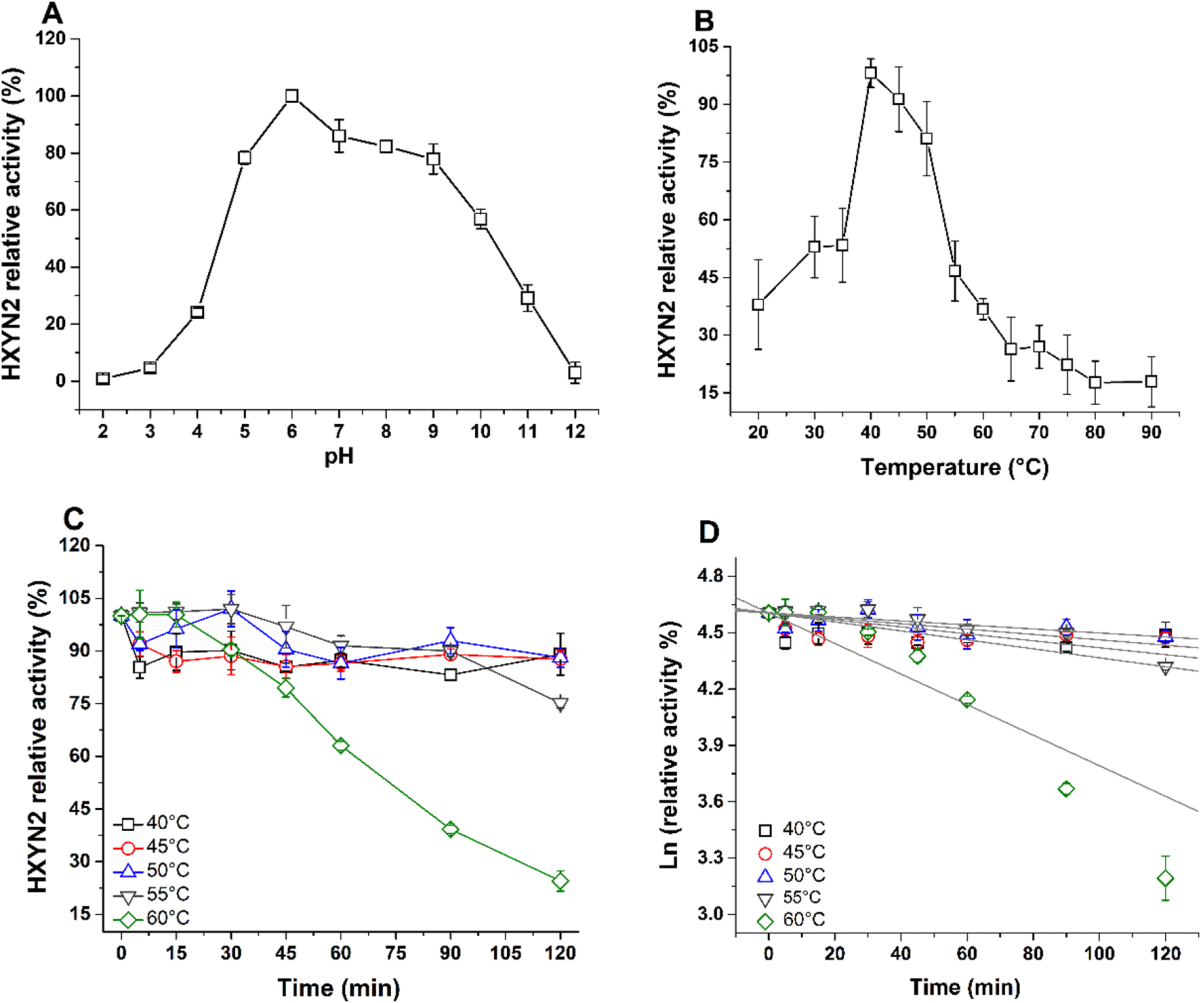
Effect of temperature and pH on HXYN2 activity and thermostability. (**A**) Relative activity of HXYN2 at pH values ranging from 2.0 to 12. (**B**) Relative activity of HXYN2 over a temperature ranging from 20 to 90 °C, at pH 6.0. (**C**) Thermostability of HXYN2 after incubation at 40, 45, 50, 55, and 60 °C for different periods up to 2 h. (**D**) First-order plot for thermal denaturation of HXYN2.

The concentration of HXYN2 employed in all enzyme assays was standardized to 400 nM according to the linear correlation between the enzyme concentrations *versus* the initial velocity (Supplementary Fig. S4). The activity of HXYN2 at pH values ranging from 5.0 to 9.0 varied between 75 and 100%, with a maximum activity occurring at pH 6.0 (Fig. 5A).

The activity of HXYN2 is greater than 70% at temperatures between 40 and 50 °C (Fig. 5B). Thereafter, the activity abruptly decreases, reaching values close to 10% after 80 °C. The thermostability assay showed that HXYN2 had lost most of its activity at 65 °C, but remained stable at 40 °C, 45 °C, and 50 °C, retaining 80% of activity after 2 h (Fig. 5C). Thermostability at these temperatures was corroborated by the half-life determined from data shown in Fig. 5D (Table 1). HXYN2 displayed a half-life over 5 h at 50 °C, in contrast to Carvalho and collaborators^19^ who observed a half-life above 3 h at 50 ºC using a different substrate. In the present study, the stability and half-life of HXYN2 increased to 11 h at 50 ºC. At 55 °C 75% of activity was retained for 2 h, although at 60 °C this activity decreased 60% after 90 min of incubation (Fig. 5C). As such, different stabilities of xylanase are likely due to other incubation conditions.

**Table 1:** Kinetic parameters of thermal denaturation of HXYN2.

Temperature (°C)	*kd* (min^−1^)	R^2^	t_1/2_ (min)
40	2.30 × 10^–3^ ± 3.90 × 10^–4^	0.90	301.20
45	1.40 × 10^–3^ ± 3.00 × 10^–4^	0.95	495.00
50	1.00 × 10^–3^ ± 3.70 × 10^–4^	–	693.00
55	1.80 × 10^–3^ ± 2.60 × 10^–4^	0.95	384.60
60	8.10 × 10^–3^ ± 1.00 × 10^–3^	0.92	85.20

*kd* thermal deactivation constant, *t*_*1/2*_ half-live.

### Hydrodynamic parameters of HXYN2 by analytical ultracentrifugation

**Figure 6:**
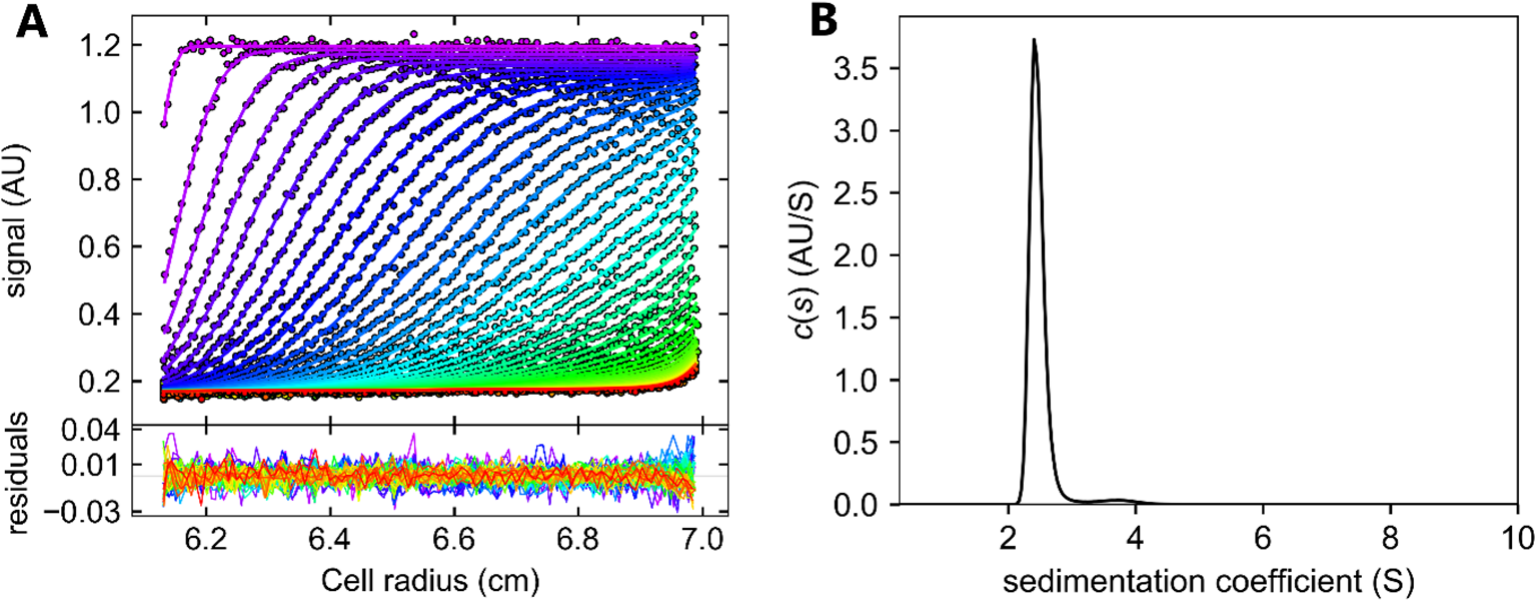
Analytical ultracentrifugation of HXYN2 in 50 mM Tris–HCl pH 7.0 and 0.15 M NaCl. (**A**) Sedimentation profile of HXYN2 (0.38 mg/mL) recorded by absorbance at 280 nm as a function of radial distance (radius) of the cell. The residual error values of the adjusted data are displayed at the bottom. The colored dots and the lines represent the collected data and the adjustment data, respectively. (**B**) Continuous distribution of HXYN2 sedimentation coefficient.

The sedimentation profile and the data adjusted for a continuous distribution of the sedimentation coefficient, corresponding to a concentration of 0.38 mg/mL, are shown in Fig. 6A,B, respectively. All other concentrations also displayed the same profile. Sedimentation data were adjusted according to the Lam equation using partial specific protein volume (0.715523), density, and buffer viscosity of 1.0058 and 0.0103119, respectively, using the SEDNTERP program. The frictional ratio of 1.18 and the rmsd of 0.0074 that were calculated from the best fit indicated a single population of monomeric molecules (90.5% of the signal). Calculations revealed a standardized sedimentation coefficient (S_20.w_) of 2.58 S and a molecular mass of 22.6 kDa. The hydrodynamic radius (2.1 nm) remained similar across the three protein concentrations. The distribution curves, c(s), of the sedimentation coefficients for the three concentrations showed only a single peak.

The sedimentation profiles at pH 4.0, 7.0 and 9.0 are shown in Supplementary Fig. S5A-C. The hydrodynamic radius and sedimentation coefficient of HXYN2 obtained from adjusted data at all pHs are shown in Table 2. The frictional ratio (f/fo) and the Stokes radius are similar in acidic, neutral and basic conditions. The sedimentation coefficients present an average value of 2.53 S, represented by a single peak (Supplementary Fig. S5D).

**Table 2:** UCA-VS parameters from adjusted sedimentation data for HXYN2 at pH 4.0, 7.0 and 9.0.

Parameters	pH 4.0	pH 7.0	pH 9.0
Concentration (mg/mL)	0.20	0.20	0.20
Rmsd	0.01	0.01	0.01
f/fo	1.20	1.16	1.20
Stokes radius (nm)	2.21	2.15	2.20
MM (kDa)	21.95	22.33	22.08
S_20, W_ (S)	2.49	2.60	2.50

### Effect of phenolic compounds on HXYN2 activity

**Figure 7:**
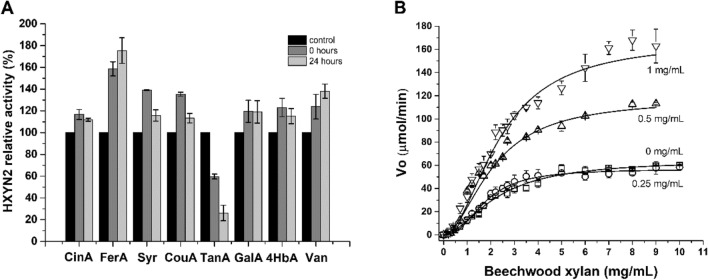
Effect of phenolic compounds on HXYN2 activity and its kinetics. (**A**) HXYN2 relative activity. The compounds comprise Cinnamic acid (CinA), Ferulic acid (FA), Syringaldehyde (Syr), p-Coumaric acid (CouA), Tannic acid (TanA), Gallic acid (GalA), 4-Hidroxybenzoic acid (4HbA), and Vanillin (Van). (**B**) Michaelis Menten curve of HXYN2 in the absence (squares—0 mg/mL FA) and presence of FA: 0.25 mg/mL (circles), 0.5 mg/mL (triangles) and 1 mg/mL (inverted triangles). Substrate concentration varied between 0.1 and 10 mg/mL.

The effect of phenolic compounds, such as FA, trans-cinnamic acid, *p*-coumaric acid, gallic acid, 4-hydroxybenzoic acid, syringaldehyde, and vanillin, showed between a 20–75% activating effect on HXYN2 activity after 0 and 24 h of incubation (Fig. 7A). At a concentration of 1 mg/mL, the phenolic compounds did not affect the DNS assay in the presence and absence of xylose. Among the compounds, FA promoted the greatest increase in enzymatic activity in comparison to the control by 60–75%. In contrast, tannic acid promoted an inhibitory/deactivating effect on HXYN2 reducing the activity to 26% and 59%, after pre-incubation with the enzyme for 0 and 24 h, respectively (Fig. 7A).

### Kinetic parameters of HXYN2 in the absence and presence of FA

The kinetic constants of HXYN2 were determined from the Michaelis–Menten plot by varying the beechwood xylan concentration under ideal conditions of temperature (50 °C) and pH (6.0) (Fig. 7B). Calculated K_m_ and V_max_ values were 2.24 mg/mL and 64.49 µmol/min, respectively. The catalytic constant (k_cat_) and catalytic efficiency (k_cat_/K_m_) were calculated as 161.22 min^-1^ and 71.97 mL/min.mg, respectively.

The Michaelis Menten kinetic parameters of HXYN2 (Fig. 7B) differed as the concentration of FA increased. In the presence of 0.25 mg/mL, there was a slight decrease in K_m_, while V_max_ was maintained. At concentrations of 0.5 and 1 mg/mL, an increase in V_max_, k_cat_ and catalytic efficiency was observed, while K_m_ was maintained. Table 3 shows the increase in k_cat_ values with accumulating concentrations of FA. In conclusion, FA does not alter the affinity of HXYN2 for the beechwood xylan substrate, but does improve the catalytic velocity and efficiency of the enzyme.

**Table 3:** Kinetic parameters of HXYN2 in the absence and presence of FA.

Parameter	K_m_ (mg/mL)	V_max_ (μmol/min)	Hill coefficient (N)	k_cat_ (min^−1^)	k_cat_/K_m_ (mL/min mg)
HXYN2	2.24 ± 0.11	64.49 ± 1.02	1.76 ± 0.06	161.22	71.97
HXYN2 + FA (0.25 mg/mL)	1.75 ± 0.05	56.71 ± 1.11	2.38 ± 0.21	141.78	81.01
HXYN2 + FA (0.5 mg/mL)	2.09 ± 0.03	117.21 ± 1.26	1.85 ± 0.96	293.02	140.20
HXYN2 + FA (1 mg/mL)	2.38 ± 0.14	167.22 ± 8.12	1.93 ± 0.05	418.07	175.65

**Figure 8:**
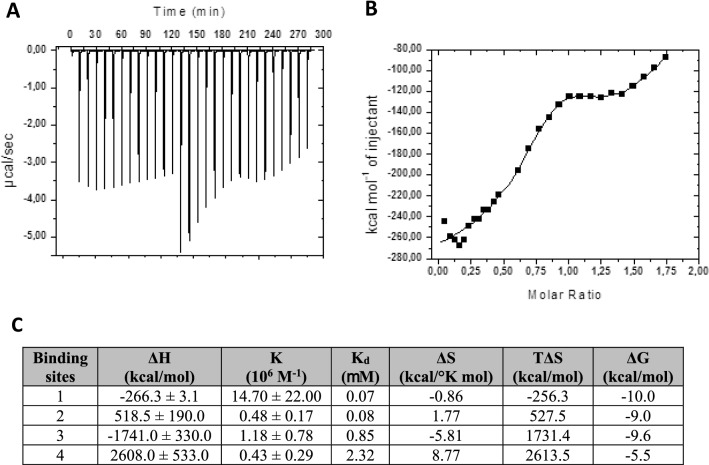
Isothermal titration calorimetry of HXYN2 with FA. **(A**) Calorimetric titration responses from successive injections of 0.1 mM FA to 9.61 µM HXYN2 into the sample cell. The titration was performed in 20 mM glycine-citrate–phosphate buffer pH 6.0 and 298 °K. (**B**) The integrated heat profile of the calorimetric titration. The solid line represents the best nonlinear squares fit to a four sequential binding sites fit model. (**C**) Thermodynamic parameters of the interaction of HXYN2 with FA. ΔH: Enthalpy of binding, K: Binding constant, K_d_: Dissociation constant, ΔS: Entropy, and ΔG: Gibbs free energy.

The thermodynamics of HXYN2 interaction with FA was also analyzed by isothermal titration calorimetry through injection of the phenolic compound into the enzyme solution. A total of four distinct sites were identified from the isotherm adjustment (Fig. 8A,B), with binding constants ranging from 0.43 to 14.7 × 10^6^ M^−1^ (Fig. 8C), equivalent to a dissociation constant (K_d_) from 2.32 to 0.068 μM.

### Fluorescence spectroscopy

**Figure 9:**
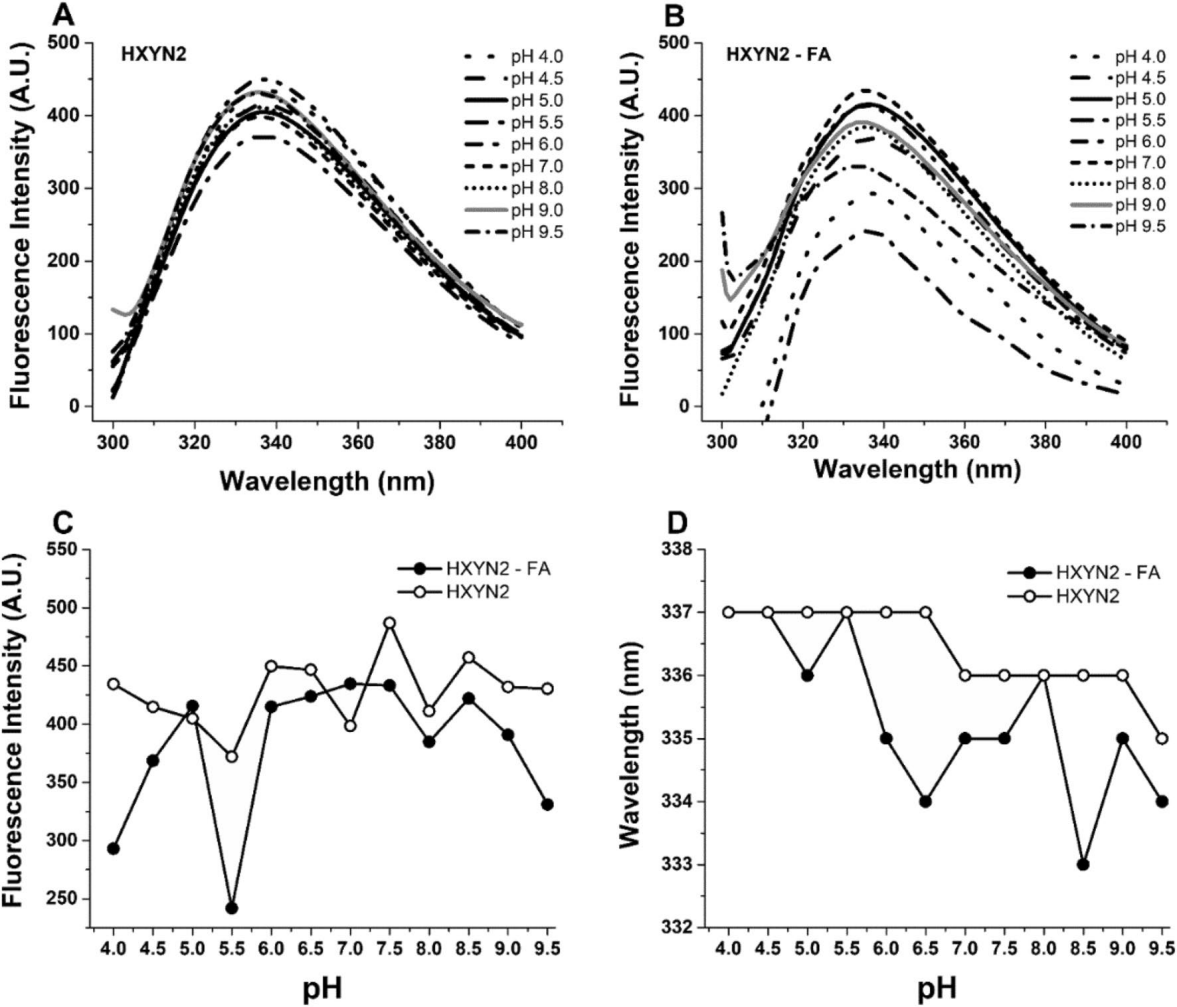
Fluorescence emission spectra of HXYN2 as a function of pHs in the absence and presence of FA. (**A**) Fluorescence spectra in the absence of FA. (**B**) Fluorescence spectra in the presence of FA. (**C**) Fluorescence intensities in the presence and absence of FA. (**D**) Maximum emission wavelength, in the presence and absence of FA.

Fluorescence spectra of HXYN2 in the absence of FA at different pHs (Fig. 9A–D) showed small changes in intensities and a slight blue shift (337 to 335) in the emission bands from acid/neutral pH (pH 4.0–7.0) to basic pH (8.0, 9.0, and 9.5). Additionally, the slight shifts were observed in the blue wavelength of the maximum emission bands at pH 6.5–7.0 and 9.0–9.5, close to the pKa of His and Tyr, respectively.

Conversely, in the presence of FA (Fig. 9B–D), intensities and displacements of the emission bands showed greater differences as a function of pH. A marked decrease in the maximum emission wavelength (between 337 and 333 nm) occurred between pH 6.0–7.5 and 8.5–9.5 (Fig. 9D), while the fluorescence intensities (Fig. 9C) over a similar range (pH 6.0–9.0) remained constant.

**Figure 10:**
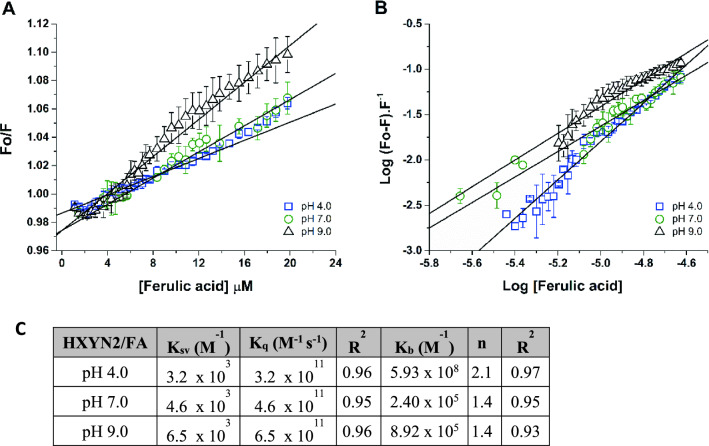
Fluorescence quenching of HXYN2 by interaction with FA. (**A**) Stern–Volmer plot of the interaction between HXYN2 and FA at pH 4.0, 7.0, and 9.0. Relative increases of fluorescence intensities at 335 nm (F/F_0_) versus FA concentration were used to estimate Stern–Volmer constants (K_sv_). (**B**) Plots of Log (F0 − F/F) versus Log (FA) for FA quenching effect on HXYN2 emission fluorescence at pH 4.0, 7.0, and 9.0. (**C**) Stern–Volmer constants (K_sv_), Bimolecular quenching constant (K_q_), Binding constants (K_b_), and binding sites (n) of HXYN2 in complex with FA at different pHs.

The interaction of FA with HXYN2 was evaluated by fluorescence quenching, in which a decrease in fluorescence intensity was observed as a function of increasing concentrations of FA. Data were analyzed according to the Stern–Volmer equation, with the linear correlation (Fig. 10A) used to estimate the Stern–Volmer constants (K_sv_) (Fig. 10C).

The K_sv_ values of the HXYN2-FA complex are of the order of 10^3^. The values of the bimolecular quenching constant, K_q_, ranged from 3.2 × 10^11^ to 6.5 × 10^11^ M^−1^ s^−1^, at acidic and basic pH (Fig. 10C). The binding constants (K_b_) and the number of binding sites for the FA molecule (n) in the HXYN2 protein, as a function of pH, were calculated from the data shown in Fig. 10B. The number of FA binding sites on HXYN2 is approximately 1 at pH 7.0 and 9.0, and 2 at pH 4.0. The binding constant is greater at pH 4.0 than at pH 7.0 and 9.0 (Fig. 10C).

### Secondary structure and structural stability of HXYN2 based on circular dichroism

FAR-UV CD spectra of HXYN2 at different pHs at 25 °C (Supplementary Fig. S6) showed a positive and negative dichroic signal at ~ 198 nm and 218 nm, respectively. A slight reduction in the dichroic signal was observed in the negative and positive bands at pH 4.0 and 9.0, as compared with those at pH 6.0 and 7.0. The low percentage of α-helix, as compared to the predominant β-sheet structures was estimated at all pHs (Supplementary Fig. S6—Table inset).

The Far-UV CD spectra of HXYN2 at temperatures ranging from 25 to 95 °C and at different pHs were recorded to monitor the thermal stability of proteins dependent on pH (Supplementary Fig. S7A–D). At pH 4.0, the dichroic bands at 218 nm were maintained close to −4350 deg cm^2^/dmol between 25 and 45 °C (Supplementary Fig. S7A). A temperature increase from 45 to 95 °C resulted in gradual decreases (−4230 to −1499 deg cm^2^/dmol) and displacements of the dichroic bands. The spectra at pHs 6.0, 7.0, and 9.0 (Supplementary Fig. S7B–D) showed similar dichroic bands at 218 nm from 25 to 55 °C, which decreased abruptly from 55 °C (~ −7000 deg cm^2^/dmol) to 65 °C (~ −2000 deg cm^2^/dmol).

**Figure 11:**
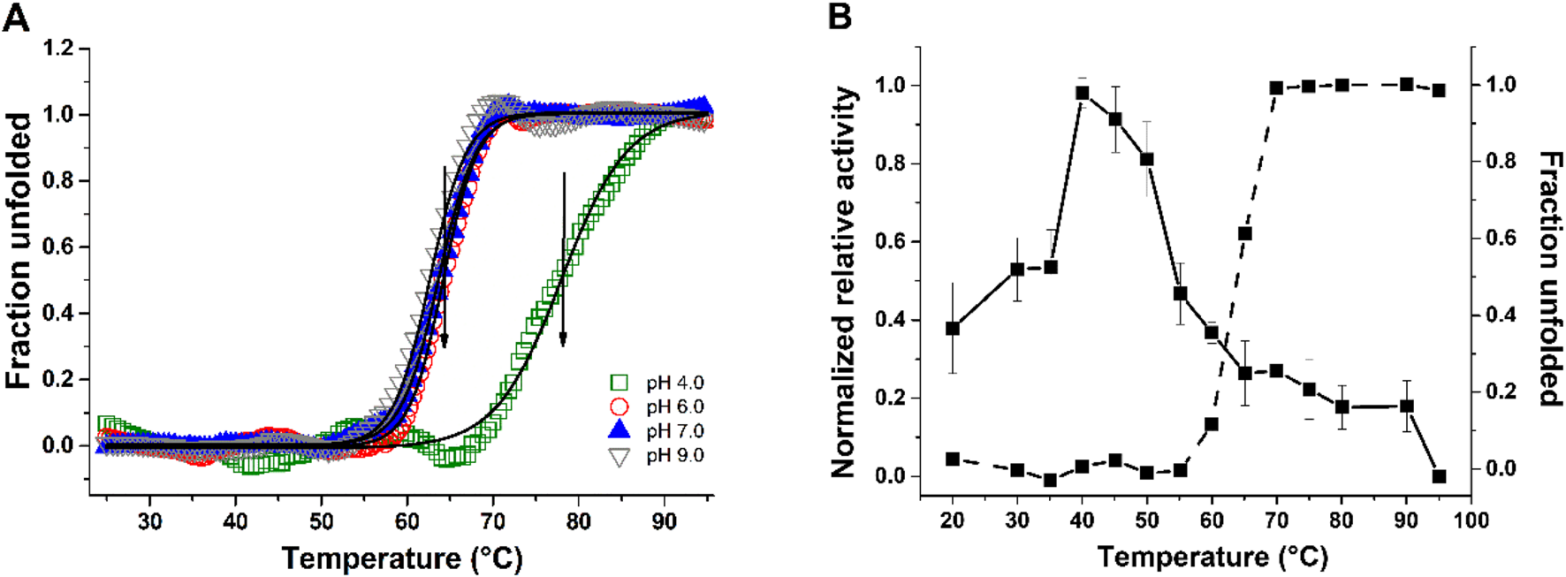
The fraction of unfolded protein (Fu) and normalized relative activity of HXYN2 as a function of temperature. (**A**) Fraction of unfolded HXYN2 (Fu) at pH 4.0, 6.0, 7.0, and pH 9.0 as a function of temperature ranging from 25 to 95 °C. The arrow indicated the T_m_ of 76.2 °C, 63.5 °C, 63.7 °C, and 62.3 °C at pH 4.0, 6.0, 7.0, and pH 9.0, respectively. (**B**) Comparison between the enzyme activity and unfolding curves of HXYN2 at pH 6.0 (solid line and dashed line square, respectively).

**Figure 12:**
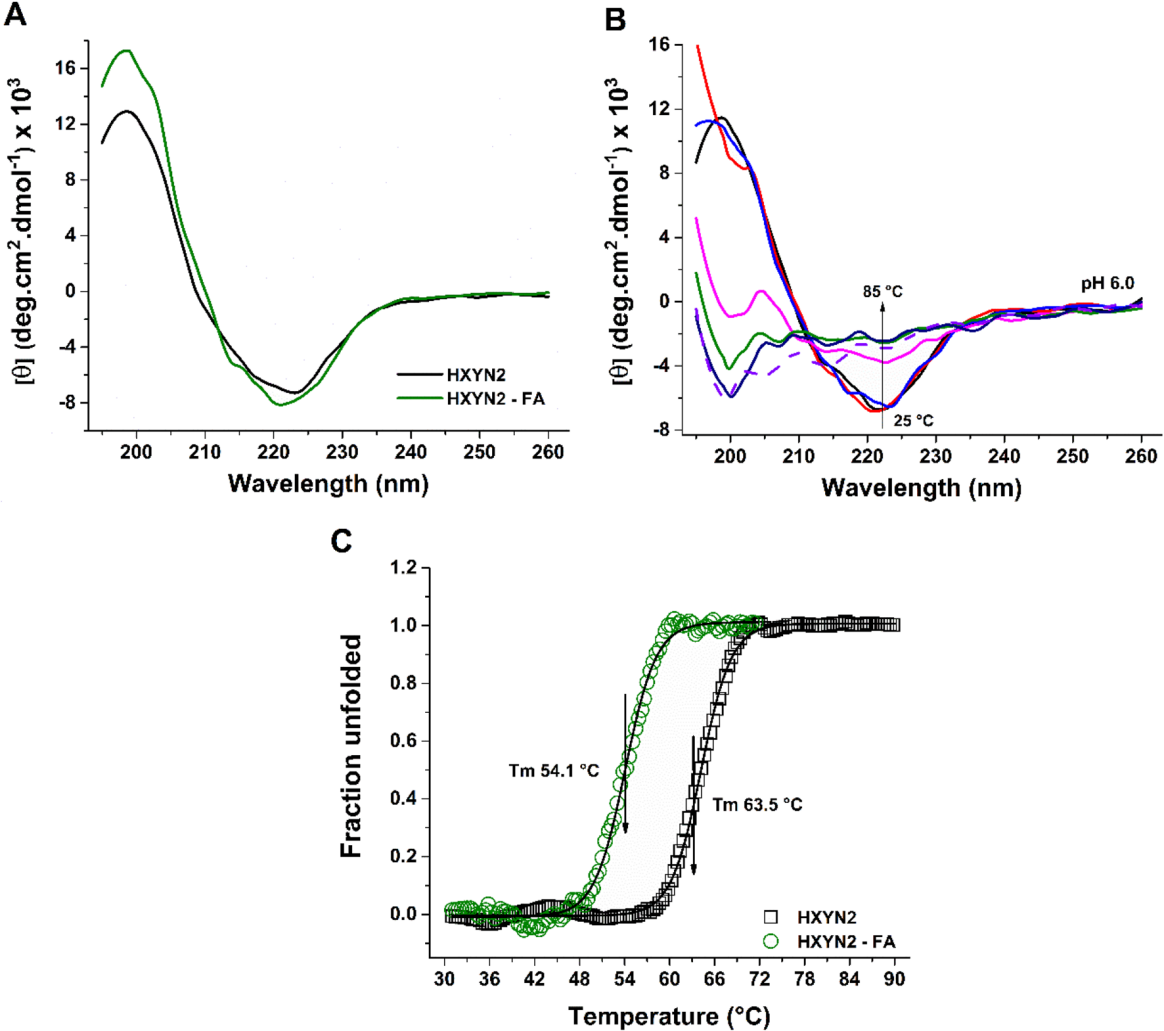
Far-UV CD spectra and unfolding curves of HXYN2 at pH 6.0, as a function of temperature and FA. **(A**) Far-UV CD spectra at 25 °C in the absence and presence of FA. (**B**) Far-UV CD spectra in the presence of FA at temperatures ranging from 25 °C (solid line) to 85 °C (dashed line) at pH 6.0. The arrow indicates the decrease in molar ellipticity with rising temperature. (**C**) Fraction of unfolded HXYN2 (Fu) at pH 6.0 in the presence (line with circle) and absence (line with square box) of FA at temperature ranging from 25 to 95 °C.

The unfolding curves at pH 4.0, 6.0, 7.0, and 9.0 showed typical transition from native to denatured states with the melting temperatures (T_m_) of 76.2 °C, 63.5 °C, 63.7 °C, and 62.3 °C, respectively (Fig. 11A). Figure 11B shows the enzyme activity and unfolding curve of HXYN2 at pH 6.0. The relative activity curve intercepts the unfolding curve in the temperature range in which HXYN2 loses activity. Far-UV CD spectra of HXYN2 in the absence and presence of FA at pH 6.0 and 25 °C showed slight differences at the positive dichroic signal at ~ 198 nm (Fig. 12A). In addition, Far-UV CD spectra of HXYN2 at temperatures ranging from 25 to 45 °C in the presence of FA (Fig. 12B) are similar. However, raising the temperature from 45 to 85 °C resulted in an abrupt decrease and displacement of the dichroic bands to approximately 200 nm. The unfolding curves showed a decrease in the T_m_ in the presence of FA from 63.5 to 54.1 °C (Fig. 12C).

### Molecular docking

**Figure 13:**
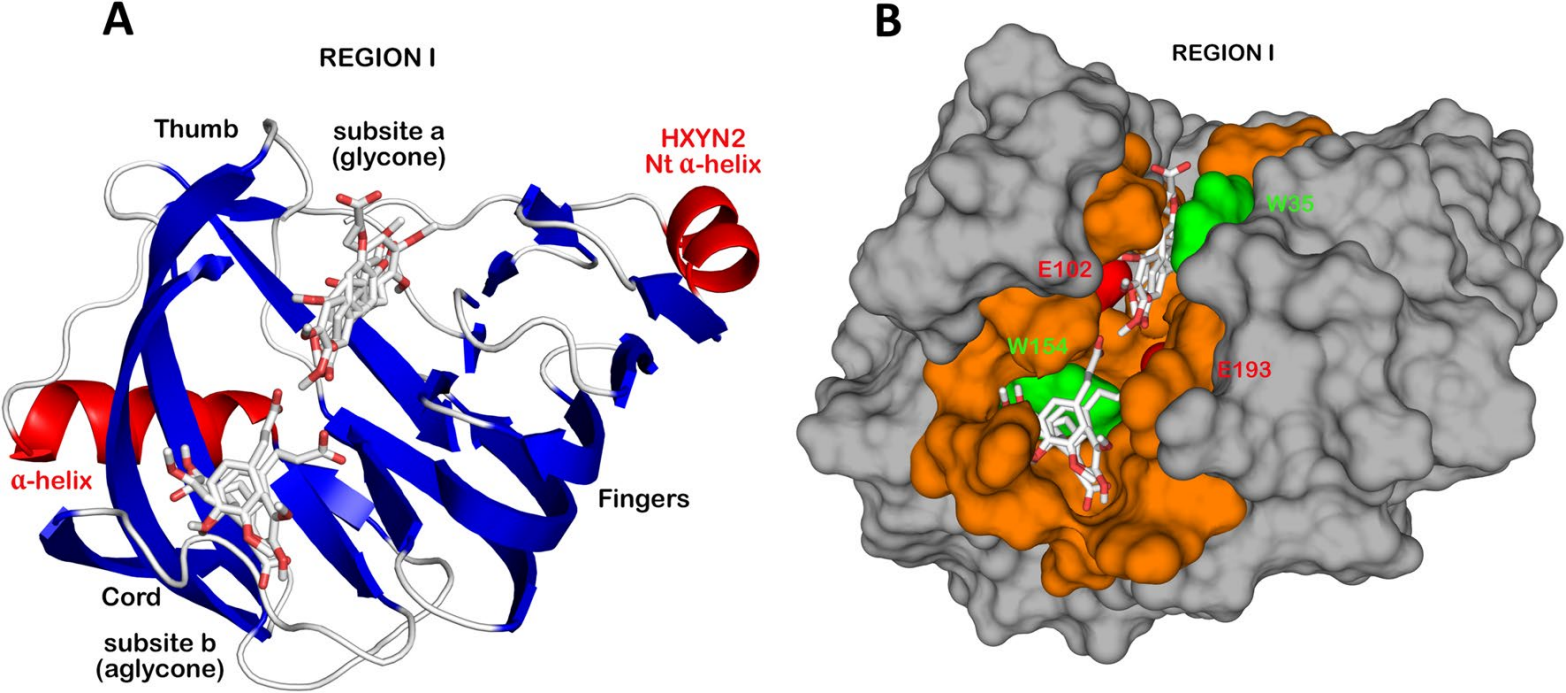
Possible FA binding sites on HXYN2 as evaluated by molecular docking. (**A**) HXYN2-FA complex showing the docking solutions in region I involving aglycone and glycone sites. FA is represented as stick format and the protein in cartoon. (**B**) Residues that interact with FA are highlighted in orange, and Trp and Glu catalytic residues are represented in green and red, respectively. FA is represented as a stick format and the protein as molecular surface. Figures were generated using *PyMol* *Molecular* *Graphics* *System* *version* *2.1.1* (http://www.pymol.org).

The structural details of the HXYN2-FA complex interface were analyzed by molecular docking. Forty-five molecular docking solutions with favorable Gibbs free energies (−3.4 to −6.8 kcal/mol) were predicted and grouped into five regions of potential interaction, each with nine docking solutions (Supplementary Table S3). The solutions (Fig. 13A,B, Supplementary Table S3, and Supplementary Fig. S8) show the most favorable free energy values for FA binding in region I involving aglycone (solutions 5, 7, 8, and 9) and glycone (solutions 1, 2, 3, 4, and 6) regions of the catalytic sites. It is important to note that some solutions clustered in region I involved at least one tryptophan residue (W35 or W154) and one or both Glu catalytic residues (E102 and E193). Additionally, the hydrophobic and van der Waals interactions with the N61, Y112, N113, P114, G115, A118, R138, Y195, and E193 catalytic residue are highlighted (Supplementary Table S3).

## Discussion

### Enzyme production and purification

Heterologous expression and purification of HXYN2 were successfully performed resulting in culture supernatant xylanase activities of 354 U/mL and 415 U/mL, respectively. Purity and absence of glycosides of the expressed protein were confirmed by the similar molecular mass of 23 kDa estimated from both SDS-PAGE and amino acid sequence prediction.

### Molecular modeling of HXYN2 structure

Currently, only two GH11 xylanase structures with branched oligosaccharides have been elucidated: the NpXyn11A-arabinoxylo-oligosaccharide complex from *Neocallimastix* *patriciarum*^11^ that was used as a template to obtain the HXYN2 structure, and the SoXyn11B-arabinoxylo-oligosaccharide and SoXyn11B-glucuronoxylo-oligosaccharide complexes from *Streptomyces* *olivaceoviridis* *E-86*^30^.

The root-mean-square deviations (rmsd) of the atomic positions for Cα (Supplementary Table S2) and the TM-score between the five HXYN2 models of 0.30–0.37 Å and 0.95–0.99, respectively, indicated very high structural similarity. The substrate-binding site and the residues potentially involved in interactions with FA were identified in the superimposed HXYN2 model with NpXyn11A and SoXyn11B. As is already known, the 4-methylglucuronic acid side chains of branched xylo-oligosaccharide substrates bind preferentially at − 3 and + 2 subsites of GH11 xylanases, while O-3-linked arabinose side chains bind at − 2 and + 2 subsites^30^. In the case of glucuronoxylan substrate, the interaction occurs with at least three consecutive unmodified xylose residues between two modified xyloses. Similarly, in the superimposed HXYN2 model, the xylose moieties at + 1, – 1, and – 2 subsites show the 2- and 3-hydroxyl groups oriented at the bottom of the cleft (Fig. 4A,B). Conversely, the two 2- and 3-hydroxyl groups of xylose at the + 3, + 2, and – 3 subsites are exposed to the solvent corresponding to the position of the arabinose branches or a glucuronic acid side chain (Fig. 4B). The interaction with xylotriose, branched with arabinofuranose and FA at C3 of xylose 2 (Fig. 4B), occurs through N61, N87, Y89, Y112, Q152, R138, W154, and Y195 residues (Fig. 4C).

Enzymatic hydrolysis of glycosidic bonds occurs via acid catalysis by a double-displacement catalytic mechanism in which a covalent glycosyl-enzyme intermediate is formed^8,32^. Based on the structural similarity of HXYN2 with GH11 xylanases and superimposed models (Fig. 4 and Supplementary Fig. S3), a similar catalytic mechanism for the hydrolysis of xylan β-1,4 xyloside bonds can be proposed. In the retained anomeric carbon configuration in HXYN2, the nucleophilic catalytic base is in close vicinity to the sugar anomeric carbon, with an average distance between the two catalytic residues of ~ 5.0–8.0 Å (7.5 + 0.7 Å in HXYN2 model). This result suggests the retention mechanism for HXYN2, in which the anomeric carbon of xylose − 1 gives rise to a product with a β-configuration similar to the substrate.

### Effect of concentration, pH, and temperature on HXYN2 activity and its thermal stability

The maximum activity of HXYN2 occurring at pH 6.0 is similar to that obtained for GH11 xylanases from *Aspergillus* *tamarii*^33^ and *Emericella* *nidulans*^34^, where pH optimal values were 5.5 and 6.0, respectively. Previous reports show that the pH optimum of GH11 xylanases depends on the amino acid in proximity to the Glu catalytic acid–base residue^11^. Therefore, enzymes with an acidic-optimal pH generally exhibit an aspartic acid close to the Glu catalytic acid–base residue, while those with basic pH-optimal exhibit asparagine^35^.

In the case of HXYN2, an N61 close to E193 suggests that this enzyme has an optimal neutral-basic pH. However, HXYN2 showed a high activity over a wide pH range (5–9) (Fig. 5), suggesting that other structural factors may also influence its enzymatic activity, which merit future investigation. For example, the ionization state of a number of charged amino acid side chains close to the catalytic Glu residues can favor the interaction of the enzyme with the substrate and catalysis. In particular, the proximity (~ 1.74 Å) between the protonated hydroxyl oxygen of Y104 and the oxygen (OE2) of E193 can promote the Glu protonation required for catalysis over a wide pH range up to the pKa of the Tyr (~ 10.07). In this context, HXYN2 loses about 20–25% of its activity in the pH range of 7.0 to 9.0, whereas in the pH range of 10.0–12.0 the loss is between 40–100%, corresponding to the pKa value of the Tyr side chain (Fig. 5A). The change in enzyme activity as a function of pH may be due to several factors, including the pKa of the catalytic residues, charge distribution in the substrate binding site and conformational changes of the enzyme. Similarly, variations in pH can alter substrate conformation and its availability for binding and enzymatic catalysis. Favorable and unfavorable pH–dependent conformations can lead to a difference in the active site hydrogen bonding network and, consequently, in the functional properties of the enzyme. As seen by fluorescence spectroscopy (data shown later), HXYN2 exhibits slight pH-dependent conformational changes that affect the distance between the catalytic residues and enzyme activity.

Previous studies have reported that a high content of arginine increases the activity of xylanases under alkaline conditions^36,37^. For example, the arginine content of GH11 Xyn11-1 xylanase from saline-alkali soil is 6.9%, much like the alkali-tolerant XynG1-3 xylanase, and much higher than 3.0% in acidic xylanases^36^. HXYN2 possesses 5.3% arginine and high activity over a wide range of pH, indicating its alkali-tolerance condition. This characteristic suggests HXYN2 as a strong candidate for biotechnological applications, such as biobleaching and biopulping processes. Furthermore, similar to other xylanases, the structural stability of HXYN2, evaluated by circular dichroism and fluorescence spectroscopy, is independent of pH in the range of 4.0–9.0 (Supplementary Fig. S6), as well as to the presence of charged amino acid residues^38^.

### Hydrodynamic parameters of HXYN2 by analytical ultracentrifugation

Analytical ultracentrifugation by sedimentation velocity (AUC-SV) assays were conducted to assess the effect of enzyme concentration on the oligomerization process and to estimate the molecular mass of HXYN2. The hydrodynamic parameters obtained from the best fit indicate that HXYN2 appears in monomer form at pH 7.0, even in the elevated concentration, without a protein–protein association. The distribution curves, c(s), of the sedimentation coefficients for the three concentrations showed only one peak (90.5% of the signal), which represents the contribution of a homogeneous single population of monomeric molecules. This result is consistent with the hydrodynamic radius since this peak corresponds to molecules with a molecular mass of approximately 23 kDa. Therefore, in these conditions, HXYN2 is in monomeric form.

The hydrodynamic parameters at pH 4.0, 7.0 and 9.0 suggest that HXYN2 remains as a monomer with a molecular mass of approximately 22 kDa. These results indicate that HXYN2 does not self-associate and the monomeric folding is maintained over a wide pH range. The increased protein concentration and change in pH do not contribute to the association of HXYN2. Therefore, HXYN2 remains in the monomeric form at all concentrations and at all pHs employed for enzymatic and structural characterization.

### Effect of phenolic compounds on HXYN2 activity

FA acid and tannic acid promoted approximately 75% and 59% activating and deactivating effect on HXYN2 after 24 h of incubation, respectively. Similarly, FA has been shown to increase the enzymatic activity of xylanases from *Emericella* *nidulands*^34^, *A.* *tamarii*^39^, using oat spelt xylan, and *A.* *terreus*^40^ using birchwood xylan as substrates, with a reduction in K_m_ value and consequently enhancing the enzyme affinity.

Tannic acid derived from lignin in plants can associate with proteins and form irreversible complexes, leading to aggregation/precipitation and an abrupt decrease in protein activity^41^. It is known that tannic acid has a strong inhibitory effect on commercial cellulases^42^, with inhibition of up to 80% on the studied fungal cellulases and β-glucosidase^43^. Recently, Ullah and collaborators^44^ reported a 17% inhibitory effect of tannic acid on GH10 xylanase from *Penicillium* *chrysogenum* using oat spelt xylan as a substrate. In contrast, Monclaro and collaborators^33^ found a 12% increase in the activity of GH11 xylanase from *Aspergillus* *tamarii* in the presence of tannic acid using birchwood xylan as a substrate. Therefore, the tannic acid´s strong inhibiting/deactivating effect on HXYN2 could be due to protein aggregation.

### Kinetic parameters of HXYN2 in the absence and presence of FA

Previously, kinetic parameters of native and recombinant HXYN2 were determined using oat spelt xylan substrate, in contrast to the beechwood xylan substrate used in the present study. As expected, the K_m_ also differed, given this different substrate, with values of 4.38 mg/mL^17^ and 7.9 mg/mL, respectively^19^. These results suggest that HXYN2 has 2.0-fold and 3.5-fold higher affinities (Table 3) for beechwood xylan than for oat spelt xylan, when compared to the native and recombinant protein, respectively.

Differences in xylanase affinity for these substrates have also been reported by other authors^45,46^. The composition and structure of beechwood xylan and oat spelt xylan substrates are mainly due to the presence of 4-*O*-methyl-d-glucuronic acid in the O_2_ and arabinofuranose in the O_2_ and/or O_3_ in xylan branches, respectively^47,48^. These differences are in agreement with the structural model of HXYN2 presented here, where the conserved R138 residue (Fig. 4B) is located close to the arabinofuranose of the oat spelt xylan substrate (less than 3 Å). Interestingly, the presence of glucuronic acid, a negatively charged monosaccharide, in the beechwood xylan structure rather than arabinofuranose in oat spelt xylan, may explain the increased affinity of HXYN2 for the beechwood xylan. This result is in accordance with the kinetic parameters of HXYN2 displayed in Fig. 7B and Table 3.

In the presence of 0.25 mg/mL FA (Fig. 7B), there is a slight decrease in K_m_, while V_max_ is maintained, suggesting no effect of FA on enzymatic activity. At concentrations of 0.5 and 1 mg/mL of FA, an increase in V_max_, k_cat_ and catalytic efficiency was observed, while K_m_ was maintained. These data indicate that FA does not modify the enzyme´s affinity for the substrate, probably due to a limited interaction at the substrate recognition sites. The increase in k_cat_ values with an accumulating concentration of FA is directly related to the improvement in catalytic efficiency of HXYN2. Therefore, FA does not alter the affinity of HXYN2 for the beechwood xylan substrate, but does improve the catalytic velocity and efficiency of the enzyme. These data suggest that the interaction of FA with amino acid residues close to the catalytic site promotes conformational changes that enhance the catalytic process, in agreement with the results obtained by fluorescence spectroscopy, as shown below. Another possibility is that the reaction is modified by hydrogen bonding and electrostatic effects, as well as by hydrophobic interactions, even without clear structural changes.

The thermodynamics of the HXYN2 interaction with FA was analyzed by isothermal titration calorimetry. The best fit of the binding isotherm occurred with four sequential binding sites. This model is useful for systems with non-identical sites, such as the binding of multiple ligands to transition metal ions and association in nanocomposites^49^. The binding processes are spontaneous, as indicated by Gibbs free energy values from −5.5 to −10.0 kcal/mol. No fewer than two independent thermodynamics stages were identified in the isotherm (Fig. 8A,B). A strongly exothermic initial stage at molar ratios below equimolarity between the enzyme and FA, and a second endothermic stage above equimolarity (Fig. 8C) were observed. The initial stage is an enthalpy-driven process associated with binding sites 1 and 3 and temperature dependent, as indicated by the negative values of enthalpy and entropy. Additionally, it can be attributed to the formation of HXYN2-FA complex by two sites with different affinities (K_d_ ~ 0.068 and 0.85 μM, respectively). Site 1 is the best for FA binding to HXYN2 with a binding constant of 14.70 × 10^6^ M^−1^ (K_d_ ~ 0.068 μM), followed by site 3 with a tenfold lower affinity (K_d_ ~ 0.85 μM). The thermodynamic parameters of binding sites 1 and 3 are in agreement with the results for the interaction of FA with bovine serum albumin, using a two-site binding model^50^. Considering that the initial stage at 25 °C is strongly enthalpic, yet entropically disadvantaged, and FA is negatively charged at pH 6 (pK = 4.58)^50^, it is expected that interactions at sites 1 and 3 occur through hydrogen bonds.

The second strong endothermic stage was assigned to binding sites 2 and 4 and characterized as a spontaneous binding process (ΔG of −5.5 and −9.0 kcal/mol, respectively), showing a similar binding constant of ~ 0.4 × 10^6^ M^−1^ (K_d_ ~ 2.5 μM), lower than those of sites 1 and 3 (Fig. 8C). As indicated by the thermodynamic parameters, binding sites 2 and 4 are enthalpically disadvantaged but entropically favored, which characterizes an entropy-driven process. This thermodynamic process indicates a classical hydrophobic interaction between FA and the enzyme at sites 2 and 4 in which water molecules are released from the protein interface with a significant reduction in the degrees of freedom of the molecules. Due to the complexity of the ITC results, thermodynamic processes involving the four interaction sites may also be associated with conformational changes in the enzyme induced by FA binding. Therefore, the ITC data suggest that the interaction of FA with HXYN2 occurs through initial and final stages as enthalpy- and entropy-driven processes, respectively, and at least one or two binding sites that involve conformational changes.

### Fluorescence spectroscopy

Fluorescence spectra of HXYN2 in the absence of FA at different pHs (Fig. 9A–D) suggest smaller conformational changes of HXYN2 at pH 5.0–9.0 than at pH 4.0, 4.5, and 9.5. This is in agreement with enzymatic activities, which were maintained in the range of 80–100% and 25–65% at these pHs, respectively. Additionally, the slight shifts in the blue wavelength of the maximum emission bands at pH 6.5–7.0 and 9.0–9.5, close to the pKa of His and Tyr, respectively, could be attributed to changes in ionization states of those residues close to tryptophan. In agreement with these results, the side chains of three His residues (H171, H178, and H185) in the HXYN2 3D model are located less than 7 Å from the W175 side chain. Similarly, W34, W68, W95, W154, and W175 are located less than 6 Å from the Y44, Y30, Y93/Y186/Y187, Y89/Y104, and Y153, respectively. Although the emission bands have not significantly changed at the optimum pH of the enzyme (pH 6.0) and in the pH range where its activity remains above 80% (pH 7.0–9.0), slight variations in fluorescence intensity were observed. These variations are typical microenvironmental changes in the tryptophan side chains that appear to be essential for enzyme activity. These data are in agreement with those reported for the GH10 endoxylanase from *Penicillium* *chrysogenum*^44^, in which the emission bands from pH 5.0 to pH 6.0 remained centered at 337 nm and shifted to 335 nm at pH 9.0, suggesting slight protein conformational changes.

Conversely, in the presence of FA (Fig. 9B–D), the intensities and displacements of the emission bands showed greater differences as a function of pH. These data suggest that the tryptophan side chains change from solvent-exposed to partially buried, although their micromolecular environment in the mentioned pH range does not change in the presence of this phenolic compound. In contrast, at pH range 4.0–5.5 and 9.5, the fluorescence intensities were abruptly altered, whilst the maximum emission wavelengths were practically maintained, when compared to the spectra of HXYN2 in the absence of this compound (Fig. 9C,D). These results indicate the tryptophan side chains at these pHs remain exposed to the solvent. However, the presence of FA promotes differences in the micromolecular environment of tryptophan residues close to the active site (W154), as suggested by the molecular docking results shown below. In this context, FA can modify this environment through hydrogen bonds and electrostatic effects, as well as through hydrophobic interactions, without evident structural changes. Considering the molecular docking and the pka value of FA (pK = 4.58)^50^, it is expected that FA is protonated and uncharged below pH 5.0, with a more favorable hydrophobic interaction π-π type with the W154. Nonetheless, at pH above 5.0, FA acquires a negative charge through deprotonation. Then, electrostatic interactions with charged residues, such as R138, and hydrogen bonds with Q117, Q152, Y89, Y112, and Y195 become predominant. The maintenance of the solvent-exposed tryptophan residues and the ionized state of the amino acid side chains close to the active site of HXYN2, in the presence of FA, could lead to a difference in the hydrogen-bonding network of the active site. Therefore, conformational changes contribute to better enzymatic efficiency of HXYN2, as shown in Fig. 7 and Table 3.

The fluorescence quenching (Fig. 10A) suggests that FA interacts with tryptophan residues exposed to the solvent and such interactions are pH-dependent. The K_sv_ values of the HXYN2-FA complex (Fig. 10C) indicate total access of this compound to tryptophan residues^51^. As mentioned above, displacement of the emission band (337 nm to 333 nm) at pH 4.0–9.5 occurred due to the ionization of FA, glutamic acid, histidines, and tyrosine side chains, mainly those close to the catalytic site, which resulted in changes in the tryptophan microenvironment (Fig. 9B–D). This result is also corroborated by the increase in K_sv_ values as a function of the increase of pH and suggests that the hydrogen bond occurs between FA and tryptophan residues. The HXYN2-FA complex showed a high K_sv_ value, indicating greater accessibility of this compound to tryptophan residues, the highest for pH 9.0 as compared to pHs 4.0 and 7.0^52^.

The index that differentiates dynamic from static quenching^52^ is the maximum value of the bimolecular rate constant of the fluorescence quenching process, K_q_, of approximately 1.0 × 10^10^ M^−1^ s^−1^. As shown in Fig. 10C, K_q_ values at acidic and basic pH are greater than the upper limit of K_q_ associated with a diffusion-controlled bimolecular dynamic quenching process. Therefore, the results indicated that FA is a static quenching agent that forms a complex with HXYN2, involving interaction with tryptophan residues^53^.

The binding constants (K_b_) and the number of binding sites for the FA molecule (n) in HXYN2, as a function pH, were pH dependent and indicate a moderate (at pH 7.0 and 9.0) to strong (at pH 4.0) affinity between the two molecules (Fig. 10C)^54^. These results indicate that FA can bind better to hydrophobic sites, in its neutral form and at a pH of 7.0 and higher, since it has strong hydrophobic regions in its structure. These results are also in agreement with the predominant types of interactions predicted by molecular docking, which occur at pHs below and above the pKa value of FA.

### Secondary structure and structural stability of HXYN2 based on circular dichroism

FAR-UV CD spectra of HXYN2 at different pHs at 25 °C (Supplementary Fig. S6) show a typical protein with predominant β-sheet structures. A slight reduction in the dichroic signal at pH 4.0 and 9.0, as compared with those at pH 6.0 and 7.0, suggest that the secondary structure contents were similar at all pHs (Supplementary Fig. S6—Table inset). The low percentage of α-helix, as compared to the predominant β-sheet structures (~ 65%), is in agreement with GH11 family xylanases deposited in the PDB, together with previous and the current HXYN2 structural models (Fig. 3)^24^. This structural organization and the slight conformational changes of HXYN2 in pH range 4.0 to 9.0 are important for the maintenance of the catalytic cleft and enzymatic activity.

The Far-UV CD spectra of HXYN2 at temperatures ranging from 25 to 95 °C at pH 4.0 exhibit the gradual decrease and shifts of the dichroic bands at 218 nm, indicating loss of secondary structure compatible with the denatured state of HXYN2. The spectra at pHs 6.0, 7.0, and 9.0 (Supplementary Fig. S7B–D) show similar dichroic bands at 218 nm from 25 to 55 °C, which decrease abruptly from 55 to 65 °C, corresponding to the loss of the β-sheet and HXYN2 in the denatured state.

The Far-UV CD spectra and unfolding curves at pH 4.0, 6.0, 7.0, and 9.0 show typical transition from native to denatured states with the melting temperature (T_m_) of 76.2 °C, 63.5 °C, 63.7 °C, and 62.3 °C, respectively (Fig. 11A, Supplementary Fig. S7B–D). The higher T_m_ at pH 4.0 indicates that HXYN2 is more stable in acidic than in neutral and basic conditions. These results corroborate the enzymatic activity of HXYN2 (Fig. 5), which shows a sharp drop between the temperatures of 55 °C and 65 °C at pH 6.0 (solid line), close to T_m_ (Fig. 11B). The relative activity curve intercepts the unfolding curve in the temperature range in which HXYN2 loses activity. It seems that more than half of the enzyme activity is lost at the point of T_m_ due to minor structural changes prior to full unfolding.

The Far-UV CD spectrum of HXYN2 in the presence of FA at pH 6.0 and 25 °C suggests a slight decrease of α-helix and an increase of β-turn and random coil contents when compared to that in the absence of FA (Fig. 12A). Additionally, Far-UV CD spectra and the unfolding curves at temperatures ranging from 25 to 95 °C in the presence of FA (Fig. 12B,C) indicated loss of secondary structure compatible with the denatured state and lower stability of HXYN2. This result is consistent with the decrease in β-sheets and increase in β-turn and random coil content, which can promote structural flexibility leading to a decrease in protein stability.

### Molecular docking

In order to predict the most favorable interactions of FA with the enzyme, docking solutions that agreed with the following experimental evidence were considered: (i) the interaction of FA with HXYN2 involves tryptophan residues; (ii) the favorable Gibbs free energies and the hydrophobic and hydrogen bond interactions are in agreement with thermodynamic parameters obtained by ITC; (iii) the enzyme activity and catalytic efficiency were increased in the presence of FA, which interacts in regions close to the catalytic site. The solutions in region I (Fig. 13) are most adequate to explain how FA interacts with the enzyme. The most favorable free energy values were for binding in glycone (solutions 1, 2, 3, 4, and 6) and aglycone (solutions 5, 7, 8, and 9) regions of the catalytic sites (Fig. 13A, Supplementary Table S3, Supplementary Fig. S8).

The glycone region at the catalytic site of GH11 xylanases is crucial for substrate binding and correct orientation^55^. As such, FA should not interact in the glycone region and substrate affinity and recognition in HXYN2 remains unchanged. In contrast, the relatively weak binding of xylose to aglycone subsites would facilitate a quicker release of the product prior to deglycosylation, with high enzyme turnover guaranteed^30,56^. Furthermore, in the two GH11 xylanase structures with branched oligosaccharide (PDB: 7DFN and 7DFO)^30^, only part of the substrate appears in complex in the glycone region, suggesting a low affinity for half of the substrate and product in the aglycone region (Supplementary Fig. S3). The 1, 2, 3, 4, and 6 FA docking solutions in the glycone region, with the highest affinity (−5.7 to −6.8 kcal/mol) (Supplementary Table S3), show that FA overlaps with xylan residues between subsites − 2 to + 2. These solutions are contrary to experimental evidence that showed the same substrate affinity of HXYN2 in the presence of FA and, therefore, were not considered.

Contrarily, the 5, 7, 8, and 9 FA docking solutions data suggest that FA interacts with greater probability in the aglycone region (subsite b) (Fig. 13A and Supplementary Fig. S8), as a non-inhibitory and non-competitive form, increasing catalytic efficiency without modifying substrate affinity (Fig. 7B and Table 3). All solutions in the + 3/ + 2 subsites of the aglycone region (solutions 5, 7, 8, and 9) (subsite b, Fig. 13A,B) showed hydrogen bonds between FA and N87, Y89, S116, Q117, S136, Q152, and W154 residues of HXYN2. Additionally, the hydrophobic interactions with the N61, Y112, N113, P114, G115, A118, R138, Y195, and E193 catalytic residue are highlighted (Supplementary Table S3).

Docking solution number 5 shows the highest affinity (−5.9 kcal/mol) (Supplementary Table S3) where FA interacts with Y89, S136, and W154 by hydrogen bonds and with the cord residues Y112, N113, P114, G115, S116, and Q117 by hydrophobic interactions (Supplementary Table S3, Supplementary Fig. S8A, and S8E). Particularly, docking solution number 5 could explain the experimental evidence, since it is the only one in which FA interacts by hydrogen bonds with W154 and is at the same time far from catalytic Glu residues and the glycone-substrate binding site (Supplementary Fig. S8A and S8E).

Additionally, the docking solutions number 5 are in agreement with the ITC data, which showed that the initial step of the interaction between FA and HXYN2 involves hydrogen bonds and an enthalpy-driven process. With greater FA concentration, the second step of the complex formation was characterized as an entropy-driven process, mainly associated with hydrophobic interactions and hydrogen bonds. Furthermore, the interactions between the enzyme and FA promote the reorganization of water molecules around both structures. Therefore, the presence of different FA-binding sites to the enzyme was considered due to the complexity of the ITC results.

Finally, FA binding at the distal end might increase product release and thus increase enzyme activity. Although the meaning of such binding has been supported by the experimental data shown here, this molecular docking requires further analysis and data, including FA binding to regions other than the active site cleft.

### Mechanism of enhancement of HXYN2-xylanase activity by FA

The xylan backbone structure contains xylose and branches of arabinose and 4-O-methyl glucuronic acid, which can be extended with additional substitutes, such as ρ-coumaric acid and FA^33^. FA is commonly found in the plant cell wall linked by ester bonds to hemicellulose and by ether bonds to lignin^57^. It is also present after pretreatment of lignocellulose biomass derived from lignin breakdown products^58^. Phenolic compounds frequently inhibit a variety of cellulolytic enzymes, such as xylanases, through enzyme conformational changes with or without involvement of the substrate-binding site^42^. In contrast, however, a number of studies have also shown that phenolic compounds, such as FA, do not inhibit the activity of xylanases from *Emericella* *nidulands*^34^, *Aspergillus* *terreus*^40^, and may even increase the enzyme activity of xylanase^33^, as described in the present study for HXYN2.

In GH11 xylanases, the glycosyl-enzyme is the intermediary in trans-glycosylation and hydrolysis reactions. During hydrolysis, a water molecule accepts the enzyme´s oligosaccharide and, in the trans-glycosylation, alcohol acts as an acceptor^27,59^. In this context, a competition between the suitable alcohol-like type acceptor and a water molecule occurs in the attack of the glycosyl-enzyme intermediate to the active site, determining the type of reaction^27^. The FA phenolic compound used in this study has alcohol groups and can act as an acceptor in trans-glycosylation processes. As the anomeric carbon reacts with the phenolic alcohol group, a decrease is expected in the enzymatic activity. However, increases in HXYN2 activity were observed here, ruling out trans-glycosylation reactions. This result was ascribed to an increase in the reaction rate without modification of substrate affinity for the enzyme (Fig. 7B). As shown, FA increased the catalytic parameters V_máx_, k_cat_, and k_cat_/K_m_ of HXYN2, without modifying its K_m_ (Table 3). Interestingly, HXYN2 in the absence and presence of FA (Fig. 7B) shows a slight deviation in Michaelis–Menten kinetics in response to increasing substrate concentration (Table 3). This kinetic behavior corresponds to positive cooperativity (n > 1) and requires the participation of multiple and distinct binding sites^60^. However, the amino acid sequence and structural similarity of HXYN2 to other GH11 xylanases, coupled with analytical ultracentrifugation results (Fig. 6, Supplementary Fig. S5), indicated that HXYN2 is a monomeric enzyme with a single catalytic site.

Cooperativity has been observed in non-Michaelis–Menten kinetics for a small number of monomeric enzymes that have only a single ligand-binding site^60^. This behavior can also be observed in the kinetics of allosteric enzymes, in which the association of a ligand in a distinct site from the active site affects the substrate hydrolysis rate. Therefore, cooperativity can occur in the absence of multiple binding sites and without macromolecular oligomerization^60^. In the case of cooperativity in monomeric enzymes with a single ligand-binding site, the Michaelis–Menten equation is insufficient to describe the enzyme kinetics. Therefore, the Hill coefficient (n) is used as a measure of the magnitude of the cooperative effect^60^.

Cooperativity in monomeric enzymes can be attributed to slow transitions between two structural forms of enzyme, in substrate binding or product release, leading to a time-dependent change in its activity. For GH11 xylanases, a conformational change has been proposed in the flexible region between two structural forms A “closed” and B “open” that accelerates the enzymatic reaction^61^. This conformational change is induced by the hydrogen bond networks between the “thumb” and “finger” regions. To form the enzyme–substrate complex, the flexible thumb directs the substrate into the active site cleft, which changes from an open to a closed shape. Therefore, flexibility of the active site cleft plays an important role in substrate binding as well as in enzymatic activity and the cooperative process. In addition, activation of cooperative monomeric enzymes has been reported, which occurs by a substrate of high molecular weight that binds slowly to the enzyme^62^. This hypothesis can also be considered in the case of HXYN2, due to the very large and branched structure of the xylan substrate used in the enzyme assays. Therefore, it is important to analyze the activation rate of this enzyme with substrates of different molecular weights.

Furthermore, as shown by fluorescence, the HXYN2-FA complex formation is dose and pH-dependent. The *Stern–Volmer* plot at pH 4.0, 7.0, and 9.0 (Fig. 10A,C), as along with the K_sv_ and K_q_ values, indicated the static mechanism of fluorescence quenching^51^, compatible with the formation of HXYN2-FA complex. These results are in agreement with those reported for the xylanase from *A.* *terreus* in complex with FA, in which the interaction of FA with tryptophan residues affects binding and/or hydrolysis of substrates yet maintains the integrity of the catalytic domain^63^. According to the HXYN2 model in complex with feruloyl-arabino-xylotriose, FA interacts with R138 and Q141 residues (Fig. 4E). However, this interaction is not established between the free enzyme and the soluble FA, but rather as part of a covalent branch in the xylan structure (Fig. 4A,B). The active site in this complex is occupied by the xylooligosaccharide substrate, which limits the conformational freedom and FA interaction in this region. Additionally, molecular docking suggested that FA interacts with greater probability in the aglycone region (Fig. 13A,B) by hydrogen bonds and hydrophobic interactions, increasing catalytic efficiency without modifying the substrate affinity (Supplementary Table S3).

### How can ferulic acid enhance HXYN2-xylanase activity?

As indicated by the docking solution number 5, FA may interact with HXYN2 in the aglycone region, which play an important role in the function of GH11 xylanases. Most of the residues of this region (Y112, N113, P114, G115, S116, Q117, and A118) (Supplementary Table S3) are located in the “cord” (Supplementary Fig. S8A and S8E). This is a long loop that connects the “fingers” with the base of the “thumb” and flanks the + 3 aglycone subsite and partially closes the active site of the xylanases^8,55^. The cord is very flexible, except in the middle, due to a proline (Supplementary Fig. S8A), which defines the proper conformation of the loop^8,24^. Although it is described as a part of the structure of GH11 xylanases, its functionality has been poorly addressed^55^. The longer cord length of BsXynA xylanase from *Bacillus* *subtilis* is responsible for an increase of its activity and enzyme turnover, while causing a reduction in the affinity for the substrates. The longer cord of the BsXynA mutant can cause steric hindrance to the substrate, impeding proper binding at the + 3 subsite that flanks the cord^55^. The molecular docking, fluorescence spectroscopy and enzyme kinetics of the HXYN2-FA complex indicated that FA binding at the subsite + 3 induces slight conformational changes in the active site without altering its affinity for xylan. However, the presence of FA in this site can induce an increase in the enzyme turnover number, thus enhancing its activity and the release of the first hydrolysis product, associated with the conformational change between the "B open" and "A closed" forms^55^.

Finally, the double displacement catalytic mechanism for HXYN2 was proposed, in which one or more products are released before all substrates bind to the enzyme. In this context, the presence of FA (docking solution number 5) can promote conformational changes that facilitate the release of the first product and the entry of water to release the second product, by transglycosylation, of the α-glycosyl intermediate covalently bound to the enzyme. Consequently, the increase in enzyme turnover promotes a faster release of the first product, overcoming an important energy barrier, and the release of the second product, leading to an increase in catalytic efficiency.

## Conclusions

In this study, the structure–function relationship of recombinant HXYN2 was demonstrated through a detailed enzymatic and structural characterization. HXYN2 showed high activity over a wide pH range (5.0–9.0), in which secondary structure contents and structural stability were maintained. Generally, bacterial xylanases are active in this pH range; in contrast, fungal xylanases have activity values in narrower ranges of acidic or basic pH^8^. Therefore, the HXYN2 fungal xylanase from *H.* *grisea* possesses unusual characteristics when compared to other xylanases from the same family. The enzymatic activity of HXYN2 shows a sharp drop between the temperatures of 50 °C and 65 °C at pH 6.0, which corresponds to the same interval in which the transition from the native to the unfolded state occurs. The effect of phenolic compounds shows that FA increases HXYN2 activity by 75% without changing its affinity for the beechwood xylan substrate; however, it increased the V_max_, catalytic efficiency and turnover number of the enzyme. Data from ITC, fluorescence quenching, and molecular docking showed that FA forms a complex with HXYN2 in the aglycone region, interacting with solvent-exposed tryptophan residues and other residues in this vicinity through hydrogen bonds (N87, Y89, S116, Q117, S136, Q152, and W154), hydrophobic, and ionic interactions (N61, Y112, N113, P114, G115, A118, R138, Y195, and E193). The binding constant values of this interaction are pH dependent and indicated a moderate (at pH 7.0 and 9.0) to strong (at pH 4.0) affinity. Altogether, the results showed that FA promoted an increase in the catalytic efficiency of HXYN2 by improving product release and conformational or even other changes without affecting the affinity of the enzyme to the substrate at the glycone site. The biochemical and structural data obtained in this work underscore the biotechnological potential of HXYN2 from *H.* *grisea* for the bioconversion of plant residues rich in FA. Furthermore, HXYN2 has biotechnological potential for synergistic use in enzymatic cocktails for degradation or bioconversion of plant biomass across a wide pH range.

## Material and methods

### Culture conditions

A *P.* *pastoris* transformant was grown in Basal Salt Medium (BSM) composed of 0.18% (w/v) citric acid, 0.002% (w/v) CaCl_2_.2H_2_O, 4.34% (w/v) (NH_4_)2HPO_4_, 0.09% (w/v) KCl, 0.05% (w/v) MgSO_4_.7H_2_O, and 100 mM potassium phosphate pH 6.0^64^. This medium was supplemented with 1.0% (v/v) glycerol and 0.5% (v/v) methanol, and *Pichia* trace mineral 1 (PMT1) salt solution composted of 0.6% (w/v) CuSO_4_.5H_2_O, 0.008% (w/v) NaI, 0.3% (w/v) MnSO_4_.H_2_O, 0.02% (w/v) Na_2_MoO_4_.2H_2_O, 0.002% (w/v) H_3_BO_3_, 0.05% (w/v) CoCl_2_, 2% (w/v) ZnCl_2_, 1.43% (w/v) FeCl_3_, 0.5% (v/v) H_2_SO_4_ and 1 mL of 4.0 × 10^–5^% biotin per liter. All reagents were purchased from Sigma (St. Louis, MO, USA).

### HXYN2 production

The *P.* *pastoris* transformant expressing HXYN2 was provided by the Fungi Biotechnology Laboratory at the Federal University of Goiás. Briefly, *xyn2* cDNA was obtained from total RNA of *H.* *grisea* *var.* *thermoidea* grown in 1.0% (w/v) SCB. Gene cloning was performed using the vector pGEM-T-Easy (Promega, Madison, WI, USA). The cDNA was inserted into the vector pHIL-D2 (Invitrogen) and *P.* *pastoris* GS115 strain was transformed with the linearized vector pHILD2-xyn2 by electroporation, following the manufacturer's instructions (Invitrogen) (unpublished data). Expression of HXYN2 was performed according to Cintra et al. (2017)^22^ and optimized as follows. *P.* *pastoris* GS115 strain expressing HXYN2 was pre-inoculated in BSM. The pre-inoculum was supplemented with 4.35 mL of PTM1 salt solution per liter of culture (Invitrogen, USA), incubated on an orbital shaker (200 rpm and 28 ºC for 24 h), then centrifuged (4000×*g*, 10 min, 25 °C). Cells were then inoculated in BSM optimized with 0.5% (v/v) methanol at 28 ºC, with constant agitation employed at 200 rpm for 16 h. Methanol supplementation was performed as follows. OD_600_ 0.5–10.0, supplement with 0.5% methanol every 12 h; OD_600_ 10.0–20.0, supplemented with 0.5% methanol every 6 h and OD_600_ > 20, supplement with 1.0% methanol every 6 h. After 60 h, the culture was centrifuged (4000×*g*, 10 min, 4 °C) and the supernatant used as a source of soluble and secreted HXYN2.

### Purification of HXYN2 and SDS–PAGE analysis

The supernatant was dialyzed against distilled water and concentrated 20 times by ultrafiltration using the 500 mL Amicon system that contains a 5 kDa cut-off polyethersulfone membrane Biomax^®^ (Millipore Corporation, Billerica, USA). Concentrated supernatant (5 mL) was applied at a flow rate of 1 mL/min to the Superdex S75 Hiload 16/600 column (Cytivia, USA), pre-equilibrated with 0.05 M Tris–HCl pH 7.0 and 0.15 M NaCl coupled to a AKTA FPLC Purifier (Cytivia, USA). Fractions of high absorbance at 280 nm were collected (10 mL) and applied onto 12% SDS-PAGE gels for qualitative analysis of purity. Gels were stained with Coomassie solution^65^ and silver nitrate^66^.

### Determination of the xylanolytic activity of HXYN2

An endoxylanase activity assay was performed on a microplate by the modified dinitrosalicylic (DNS) method^67^ using 1.0% (w/v) beechwood xylan (Sigma, St. Louis, MO, USA) in 0.05 M sodium citrate pH 4.8 as a substrate. DNS reagent contained 0.71% (w/v) 3,5-dinitrosalicylic acid, 20.4%, (w/v) potassium sodium tartrate, and 0.55% (w/v) sodium metabisulphite prepared in 1.32% (w/v) sodium hydroxide. Assays were performed in triplicate, adding 90 μL of the xylan substrate solution and 10 μL of the enzymatic extract, with the mixture incubated at 50 °C for 5 min. Reactions were stopped by thermal shock in an ice bath for 5 min. A total of 150 μL of DNS was then added and samples incubated at 96 °C for 5 min. Reactions were again stopped using an ice bath and reducing sugars were measured at 540 nm in SpectraMax® (Molecular Devices, San José, CA, USA). Absorbance values were converted to an international enzymatic unit (IU), where 1 IU represents 1 μmol of reducing sugar released per minute, and normalized to IU per mL, using a standard curve obtained with xylose (Sigma, St. Louis, MO, USA) at concentrations from 0.1 to 8 mg/mL.

### Molecular modeling and docking studies

The HXYN2 structure was modeled by homology using the trRosetta algorithm, a recent, rapid and accurate protein structure prediction site (https://yanglab.nankai.edu.cn/trRosetta)^68^. Multiple alignments between HXYN2 and templates were performed using the MEGAv7 program^28^. Five models were built based on homologous templates and optimized by energy minimizations, distance, and orientation constraints predicted by a deep neural network. The root-mean-square deviation (rmsd) of atomic position values between models and templates were calculated using the *PyMol* *Molecular* *Graphics* System (http://www.pymol.org), with figures prepared using the same program. Protein structural similarities were calculated using the TM-score parameter (https://zhanggroup.org/TM-score/). Interactions in the complexes were calculated using the LIGPLOT^+^ v.2.2.4 program (https://www.ebi.ac.uk/thornton-srv/software/LIGPLOT/references.html)^31^. Parameters of molecular modeling, such as resolution, confidence, coverage, and sequence identity, were considered that predict the best model. Molecular docking prediction between model 1 of HXYN2 and FA (PDB: 3NX2)^69^ were performed using the CB-Dock automatic method with the AutoDock Vina program (http://cao.labshare.cn/cb-dock/)^70,71^. Parameters rmsd lower bound (rmsd/lb) and rmsd upper bound (rmsd/ub) were considered to provide a better ranking of docking solutions. These parameters indicate how atoms are matched in the distance calculation and how each atom matches in one conformation with itself and in the other conformation, ignoring any symmetry, respectively. Additionally, amino acid residues involved in hydrogen bonds and hydrophobic interactions and the most energetic position of the ligand (kcal/mol) were also considered.

### Biochemical characterization of HXYN2

#### Effect of enzymatic concentration on biochemical assays

The molar extinction coefficient of HXYN2 (ε_280nm_) was determined from A_280nm_ *versus* protein concentration (mg/mL) curve determined by the Lowry method^72^. Enzymatic kinetics assays were performed in triplicate with HXYN2 at concentrations of 100, 200, 300, 400, 500, and 600 nM, incubated with 1% beechwood xylan as a substrate for 0, 15, 30, 45, 60, 90, 120, 150, 180, 240, and 300 s to obtain the initial velocity (V_0_). The product was quantified by DNS method^67^. Reducing sugars were estimated from A_540nm_ measurement using a SpectraMax^®^ (Molecular Devices, San José, CA, USA) reading plate. Values of V_0_ were calculated from the initial slope of the adjusted kinetics curves using the Origin 8 program (OriginLab Corporation, Northampton, MA, USA). The value of each V_0_ was converted into enzymatic activity, where 1 IU represents 1 μmol of reducing sugar released per minute and normalized to IU per mL.

#### Effect of pH, temperature on enzyme activity and thermal stability

The optimal pH of purified HXYN2 (400 nM) was determined by incubating the enzyme with 1% (w/v) beechwood xylan (Sigma, St. Louis, MO, USA) at pH values ranging from 2 to 12 using different buffers: 0.02 M citric acid pH 2.0–6.0; 0.02 M glycine pH 7.0–9.0; and 0.02 M monosodium sodium phosphate pH 10.0–12.0. The optimum temperature was determined by measuring enzyme activities at temperatures ranging from 20 ºC to 90 ºC for 10 min, at pH 6.0. Thermal stability was determined by measuring the residual enzyme activity after pre-incubation of HXYN2 without and following addition of substrate at 40, 45, 50, 55, and 60 ºC. Enzyme activity assays were performed at pH 6.0 and a temperature of 50 °C for 5 min for aliquots collected after 5, 15, 30, 45, 60, 90, and 120 min of incubation at each temperature of 40, 45, 50, 55, and 60 °C. The thermal deactivation rate constant (*kd*) was calculated using the linear fit model^73^ from data as a function of time, using Eq. (1):

1 $$\mathrm{ln}\,A{t}_{0}=\mathrm{ln}At-kd*t$$

where, *At*_*0*_ is the enzyme activity of the initial state (U/mL), *At* is the enzyme activity of the final state (U/mL), *kd* is the thermal deactivation constant, and *t* is the incubation time of the enzymatic solution (min). The half-life (t_1/2_) of the enzyme was determined using Eq. (2):

2 $${t}_{1/2}=\mathrm{ ln}\,0.5/kd$$

#### Effect of phenolic compounds on enzymatic activity

The effects of phenolic compounds (FA, *ρ*-cumaric acid, vanillin, cinnamic acid, syringaldehyde, gallic acid, 4-hydroxybenzoic acid, and tannic acid) on the inhibition and deactivation of HXYN2 were determined at 25 °C. Enzyme activities were estimated after the pre-incubation of the enzyme for 24 h with these compounds, each at a concentration of 1 mg/mL^44^. Relative enzymatic activity was calculated considering the highest activity as 100% in the absence of phenolic compounds. All experiments were performed in triplicate, with data analyzed using the Origin 8 program (OriginLab Corporation, Northampton, MA, USA).

#### Isothermal titration calorimetry (ITC)

HXYN2 interaction with ferulic acid (FA-IUPAC: 3-(4-hydroxy-3-methoxy-phenyl-prop-2-enoic) was evaluated by ITC using the MicroCal VP-ITC calorimeter (GE Healthcare Corporation, Chicago, IL, USA). Experiments were conducted using 9.6 µM of HXYN2 with buffer in the cell, and 0.1 mM of FA in the syringe. FA was prepared in a buffer by dilution from stock solution at 50 mg/mL in 95% ethanol. Measurements were performed in a cell filled with 1.44 mL of protein in 20 mM glycine-citrate-phosphate buffer pH 6.0 at 25 °C (298 °K). Titration of HXYN2 was conducted under constant stirring (307 rpm) using the rotating stirrer-syringe with an initial delay of 100 s and 29 successive injections at 0.5 µL/s. An initial spacing setting of 500 s and an initial injection of 2 µL were employed. The following 12 injections of 5 µL were performed with an interval spacing of 600 s. The final 16 injections comprised 10 µL, with an interval spacing of 600 s.

Control experiments were performed by injecting FA into HXYN2 in the same buffer. The heat released after each injection was calculated from the raw data by integration of the peaks after subtraction of the baseline. The binding isotherms were fitted to different models using the software ORIGIN v7 (OriginLab, Northampton, MA, USA) provided by MicroCal. The best fit was determined using only two parameters, namely the binding constant (K) and enthalpy (H) on each site, following selction of the total number of sites. Values for the binding constant (K_a_), binding enthalpy (ΔH), entropy (ΔS), and the number of FA molecules involved in the binding (N) were obtained following the best fit determination. Changes in Gibbs free energy (ΔG) were calculated by Eq. (3):

3 $$\Delta G=-RT\,\mathrm{ln}\,{K}_{a}=\Delta H-T\Delta S$$

#### Kinetic parameters

The kinetic parameters K_m_, V_max_ and k_cat_, for HXYN2 (400 nM) were calculated in triplicate from the adjusted nonlinear Michaelis–Menten curve as a function of the increase in the concentrations of beechwood xylan (0.5 to 9.0 mg/mL), using the Origin 8 program (OriginLab Corporation, Northampton, MA, USA). Assays were performed at 60 s, 90 s, 120 s, 150 s, and 180 s in order to obtain the initial velocity for each concentration of substrate, at pH 6.0 and 55 °C. The kinetic parameters were also determined for HXYN2 in the presence of FA (0.25, 0.5, and 1.0 mg/mL), as described above.

### Structural characterization of HXYN2

#### Fluorescence spectroscopy assays

Fluorescence measurements were performed in the presence and absence of FA, using the FP6500 Spectrofluorometer (Jasco Analytical Instruments, Tokyo, Japan) coupled with a Peltier system Jasco ETC-273T (Jasco Analytical Instruments, Japan) for temperature control. Conformational changes of HXYN2 were analyzed at a protein concentration of 0.04 mg/mL and 23.68 µM FA, in 25 mM sodium acetate pH 4.0–6.0, 0.25 M Tris–HCl pH 6.5–9.0, and 0.25 M glycine pH 9.5. The fluorescence emission spectra were recorded at 25 °C in the range of 300 to 400 nm after excitation of tryptophan at 295 nm, with excitation and emission slits at 5 and 10 nm, respectively. Data intervals of 1 nm, gain medium, response time of 2 s, and a scanning speed of 200 nm/min were used with three averaged spectra accumulations. Fluorescence intensity background and dilution were corrected.

HXYN2 in complex with FA was investigated by fluorescence quenching as a function of pH. Trp and Trp-Tyr emission measurements were performed at 25 °C, in triplicate, after excitation at 295 nm and 275 nm, respectively. Protein fluorescence emission was recorded in the range of 300 to 450 nm. A path length quartz cuvette of 1 cm and excitation and emission slit width of 5 nm were employed. Data intervals of 1 nm, gain medium, a response time of 2 s, and a scanning speed of 50 nm/min were used with two averaged spectra accumulations. Fluorescence quenching of 0.43 µM HXYN2 with 0 to 23.68 µM FA in 95% ethanol and 0.05 M of sodium acetate buffer at pH 4.0 and 0.05 M of Tris–HCl at pH 7.0 and pH 9.0 was performed using a Jasco ATS-443 Automatic titration (Jasco Analytical Instrument, Tokyo, Japan). The fluorescence intensity background and dilution were corrected. The Stern–Volmer linear regression (Eq. 4) of the relative increase of fluorescence intensity at 334 nm (F/F_0_) *versus* FA concentration was used to describe the fluorescence quenching of Trp accessible to the quencher^52^:

4 $${F}_{0}/F=1+Kq*{\tau }_{0}\left(Q\right)=1+{K}_{SV}\left(Q\right)$$

where F and F_0_ are the fluorescence intensities at 334 nm, in the presence and absence of FA, respectively, Q is the concentration of the quencher, and K_sv_ is the Stern–Volmer constant. K_sv_ can be written as K_sv_ = K_q_ τ_0_, where K_q_ is the bimolecular quenching constant and τ_0_ is the lifetime of the fluorophore in the absence of the quencher (τ_0_ is 10^−8^ s)^52^.

The thermodynamic equilibrium-binding constants (K_b_) for HXYN2-FA interaction at different pHs and the number of FA binding sites (n) on HXYN2 were calculated from a double logarithmic plot, according to Eq. (5)^74^:

5 $$\mathrm{log}\left[\left({F}_{0}-F\right)/F\right]=\mathrm{log}{K}_{b}+n\mathrm{log}(Q)$$

where K_b_ is the binding constant and *n* is the number of binding sites per HXYN2. The values of *n* and K_b_ were obtained from the slope and the y-intercept of these plots.

#### Secondary structure and structural stability of HXYN2 based on circular dichroism

Secondary structure content and the structural stability of HXYN2 were evaluated by Circular Dichroism (CD) using the Spectropolarimeter J-815 (Jasco Analytical Instruments, Tokyo, Japan) equipped with a Peltier temperature control system connected to a water pump (Jasco Analytical Instruments, Tokyo, Japan). Assays were performed with the protein at a concentration of 0.15 mg/mL at pH 4.0, 6.0, 7.0, and 9.0 in the respective buffers: sodium acetate, Bis–Tris, and Tris–HCl at a final concentration of 5 mM. A quartz cuvette of 0.1 cm path length was used to record the Far-UV CD spectra in the range of 190 to 260 nm at 25 °C. Data pitch of 0.2 nm, standard sensitivity, a band width of 1.71 nm, digital integration time of 1 s, and a scanning speed of 100 nm/min were used in continuous scanning mode. CD spectra were obtained from the average of ten consecutive readings and the subtraction of the dichroic signal values from the buffer spectrum. The ellipticities (mdeg) were converted into molar ellipticity (θ) based on the average molecular mass per residue of 115 Da. The secondary structure content at different pHs were estimated from the spectra adjusted using the Bestsel deconvolution program (http://bestsel.elte.hu/)^75^.

Thermostability assays of HXYN2 were performed at pH 4.0, 6.0, 7.0 and 9.0 at 218 nm, corresponding to the structured region of β-sheet, with temperature ranging from 25 to 95 °C and data collection of 0.2 °C/min. In addition, the CD spectra were recorded at a step size of 0.2 nm at intervals of 10 °C using the same parameters described above. Thermal denaturation curves were plotted considering the molar ellipticity values (θ) versus temperature^76^. The unfolded status of the protein (Fu) was calculated by Eq. (6):

6 $${F}_{u}=\left(yN-y\right)/\left(yN-yU\right)$$

where, yN and yU represent the values of y (into molar ellipticity) characteristic of the folded and unfolded states, respectively. The normalized unfolding curves were adjusted using the Boltzmann equation and the Origin 8.1 software (OriginLab Corporation, Northampton, MA, USA) from which the melting temperature (T_m_) was estimated.

The effect of FA on the secondary structure content of HXYN2 was also investigated by CD. Assays were performed with protein at concentration of 0.15 mg/mL in 5 mM Bis–Tris pH 6.0 and FA at concentration of 60 µM. A quartz cuvette of 0.05 cm path length was used to record the Far-UV CD spectra in the range from 260 to 190 nm at 25 °C. Thermostability assays of HXYN2 in the presence of FA at pH 6.0 were also performed, using the same parameters described above.

#### Analytical ultracentrifugation analysis

Analytical ultracentrifugation by sedimentation velocity (AUC-SV) were performed at 42,000 rpm on the Analytical Ultracentrifuge ProteomeLab XLA/XL-I (Beckman Coulter, Brea, CA, USA), equipped with a titanium rotor An-60 Ti of four positions (Beckman Coulter, Brea, CA, USA) and a UV–visible optical absorption device. Assays were performed using HXYN2 in 0.05 M Tris–HCl pH 7.0 and 0.15 M NaCl, at concentrations of 0.10, 0.20 and 0.38 mg/mL. An analysis in 0.05 M sodium acetate pH 4.0 and 0.05 M Tris–HCl pH 9.0 at a protein concentration of 0.20 mg/mL was also performed. The partial specific volume of the protein (υ), the density (ρ) and the viscosity (η) of the solution were estimated using the SEDENTERP software (http://bitcwiki.sr.unh.edu/). The frictional ratio (f/f0) was considered as a parameter to be determined by the program. The collected radial scans were analyzed using the continuous distribution analysis model of the sedimentation coefficient (c(s)) by the SEDFIT v14.7 software.

## Supplementary Information

Supplementary Information.

## Data Availability

The datasets used and/or analyzed during the current study available from the corresponding author on reasonable request.
