# Supplementary material for: Structural and biochemical analysis reveals how ferulic acid improves catalytic efficiency of Humicola grisea xylanase
Source: Sci Rep. 2022 Jul 6;12:11409. doi: 10.1038/s41598-022-15175-w (PMC9259647; doi:10.1038/s41598-022-15175-w)
Supplement: Supplementary file 1 — Supplementary Information. [file 41598_2022_15175_MOESM1_ESM.docx]

Supplementary File

Scientific Reports

**Structural and biochemical analysis revealed how ferulic acid improves catalytic efficiency of *Humicola grisea* xylanase**

Izadora Cristina Moreira Oliveira^a1^, Aisel Valle Garay^a1^, Amanda Araújo Souza^b^, Napoleão Fonseca Valadares^a^, João Alexandre Ribeiro Gonçalves Barbosa^a^, Fabrícia Paula Faria^c^, Sonia Maria Freitas^a*^

*^a1^ Biology Institute, Department of Cell Biology, Laboratory of Biophysics, University of Brasília (UnB), Quadra 604, Asa Norte, Bloco J 1° andar, Brasília, DF, 70910-900, Brazil.*

*^b^Brazilian Biosciences National Laboratory (LNBio), National Center for Research in Energy and Materials (CNPEM), Campinas 13083-970, SP, Brazil*

*^3^Biological Sciences Institute, Department of Biochemistry and Molecular Biology, Federal University of Goiás, Goiânia, Goiás, 74690-900, Brazil*

* Corresponding author at: *Biology Institute, Department of Cell Biology, Laboratory of Biophysics, University of Brasília (UnB), Quadra 604, Asa Norte, Bloco J 1° andar, Brasília, DF, 70910-900, Brazil.*

E-mail address: nina@unb.br (S.M. Freitas).

^1^These authors contributed equally to this work

***Purity of HXYN2 analysed by SDS-PAGE***

**B**

**A**


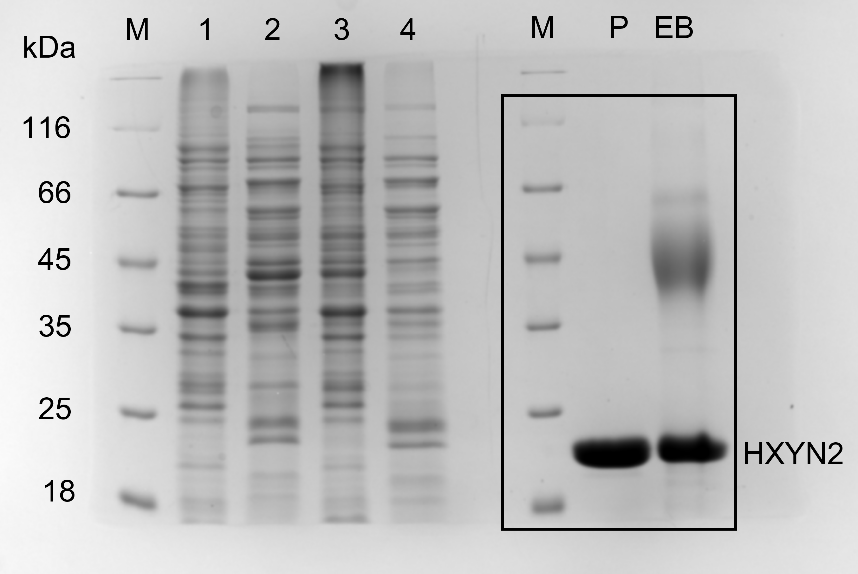

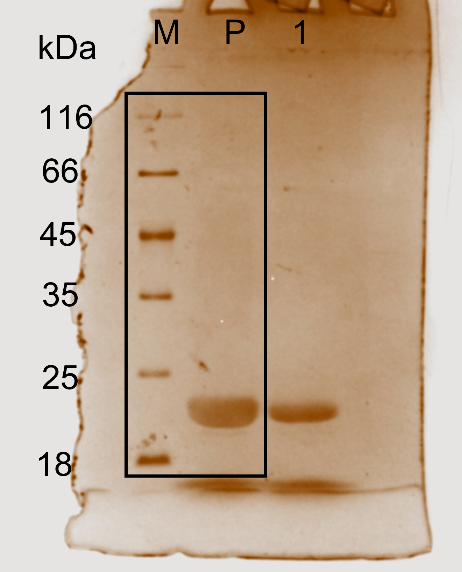


**Supplementary Fig. S1.** SDS-PAGE (12%) showing samples of expression and purification of HXYN2 (lines P and EB) and samples of expression of another protein not related to the manuscript (lines 1, 2, 3, and 4) that were opportunely applied on the same gel to use the same electrophoretic run. Lanes M, P and EB were cropped and are shown in Fig.1 of the main text. (**A**) SDS PAGE (12%) staining with Coomassie blue showing expression of HXYN2 in *Pichia pastoris* (EB), HXYN2 purified and concentrated 5X (P), and protein molecular weight markers (M). (**B**) SDS PAGE (12%) stained with silver nitrate, showing protein molecular weight markers (M), HXYN2 purified and concentrated 5X (P), and fraction of purified HXYN2 (1).

***Determination of the molar extinction coefficient***

The molar extinction coefficient (ε_280nm_) of HXYN2 was determined by the linear correlation of absorbance at 280 nm and protein concentrations by the Lowry method (Supplementary Fig. S2), using bovine serum albumin (BSA) as the standard protein. Six dilutions of HXYN2 were considered with the absorbance at 280 nm varying between 0.05 and 1.15 mg/ml. The absorbance values were converted to concentration using the ε_280nm_ of BSA estimated from the calibration curve. The ε_280nm_ of HXYN2 was calculated from the linear fit of A_280nm_ *versus* protein concentration using Origin 8 program (OriginLab Corporation, Northampton, MA, USA).








**Supplementary Fig. S2.** Determination of the molar extinction coefficient of HXYN2. (**A**) Absorption spectra of HXYN2 at different concentrations: 0.04 (solid black), 0.05 (dash black), 0.10 (solid dark gray), 0.20 (solid gray), 0.38 (solid light gray) and 0.47 (dot gray) mg/mL. (**B**) Linear correlation (R^2^=0.996) of the HXYN2 concentration determined by the Lowry method^1^ and its absorbance at 280 nm.

***The substrate interface analysis and identification of catalytic residues of the HXYN2 model with GH11 xylanase SoXyn11B (PDB: 7DFN) and with 4-o-methyl-alpha-D-3-glucuronopyranosyl xylotetraose (PDB: 7DFO)***


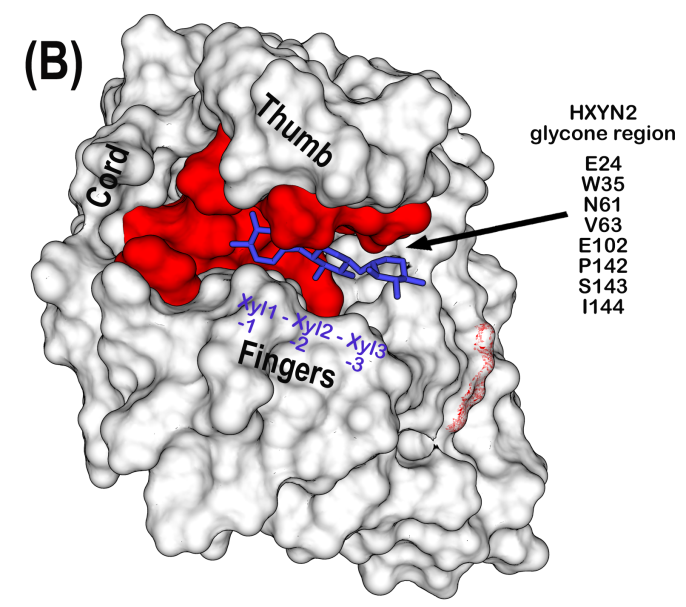

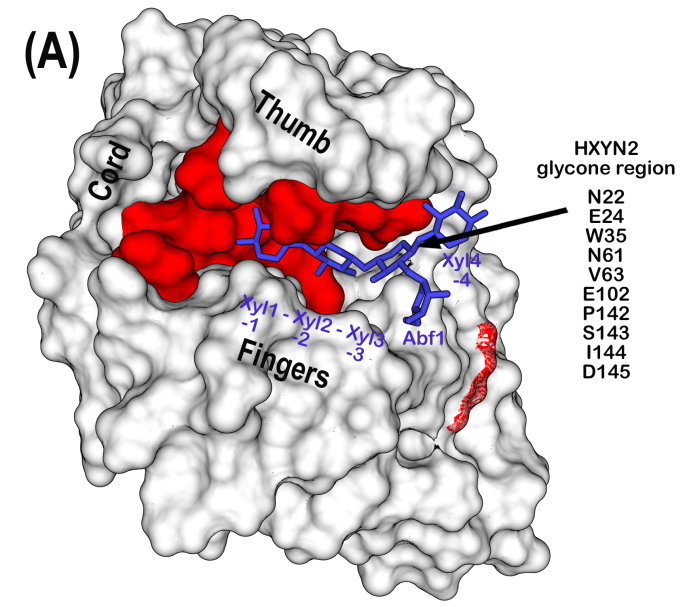


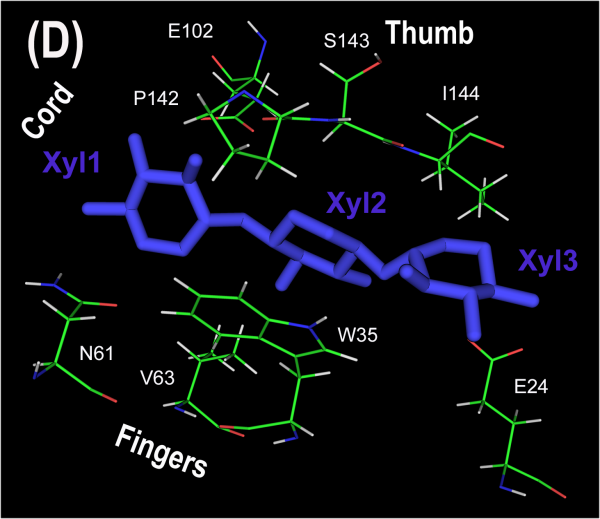

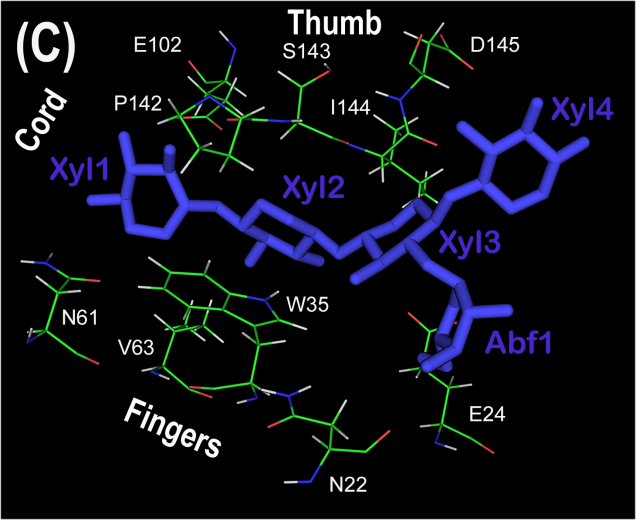


**
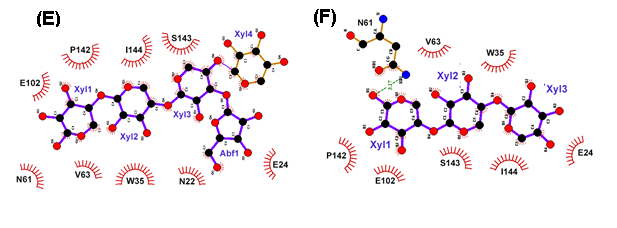
**


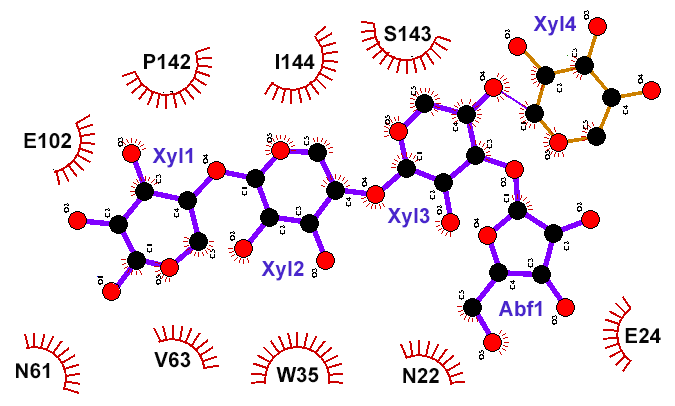

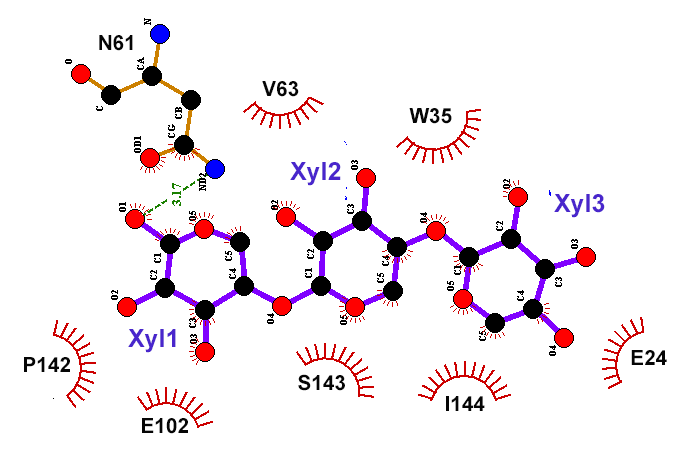


**(F)**

**(E)**


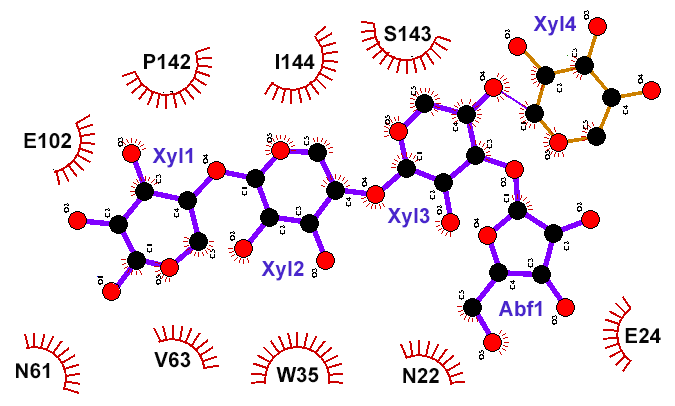

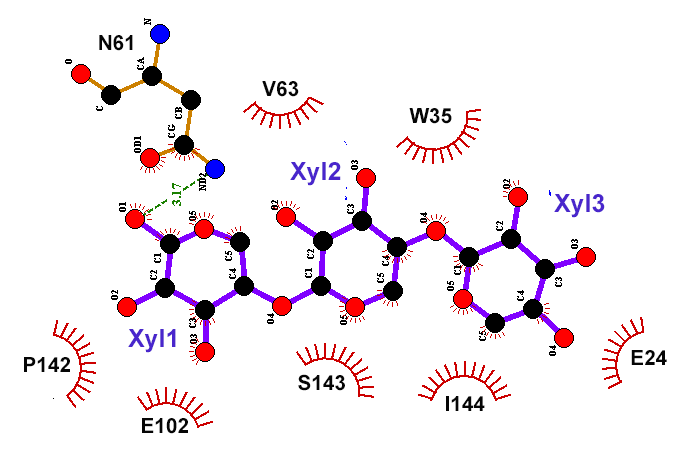


**(F)**

**(E)**


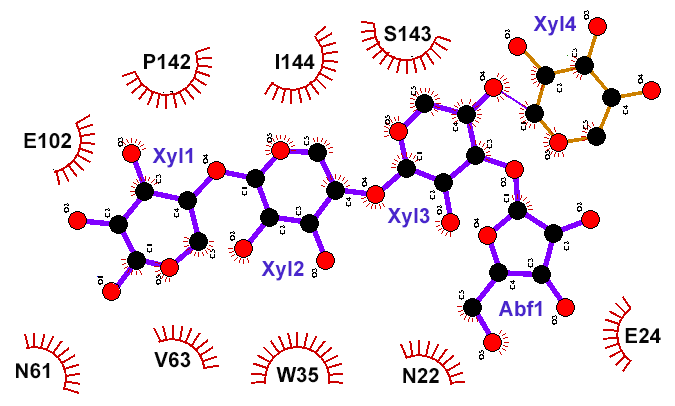

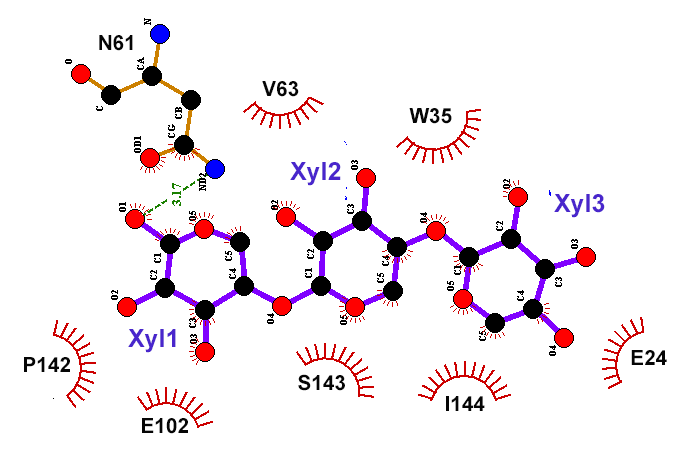


**(F)**

**(E)**


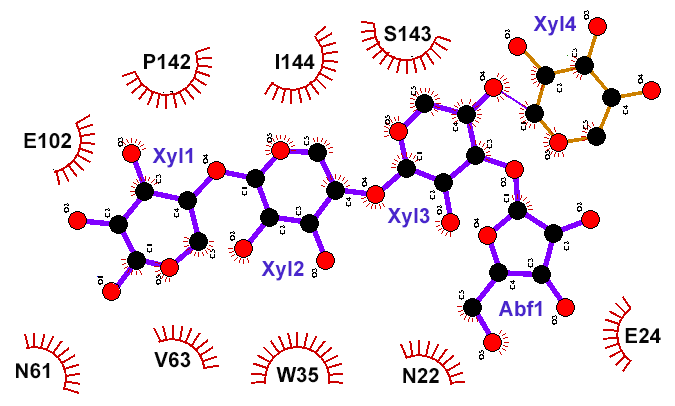

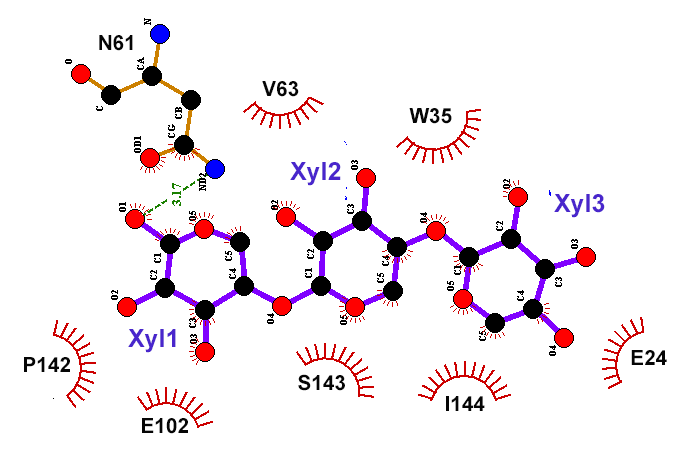


**(F)**

**(E)**


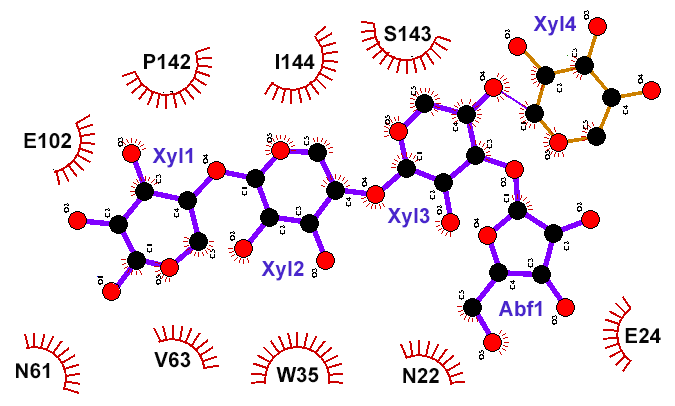

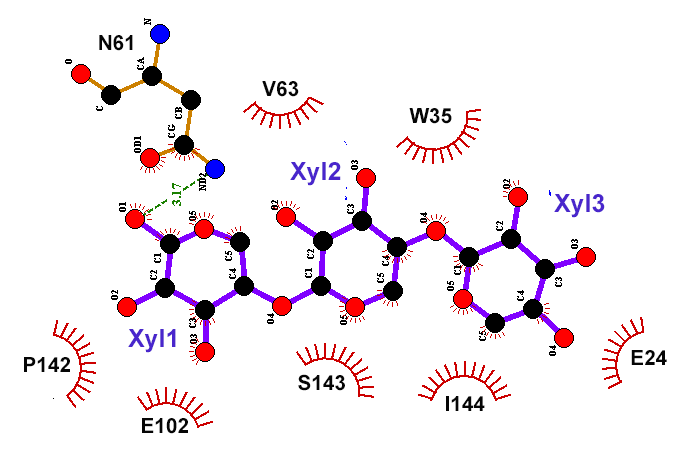


**(F)**

**(E)**


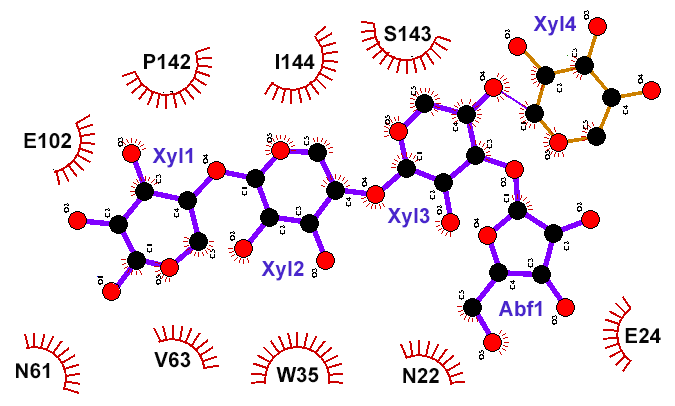

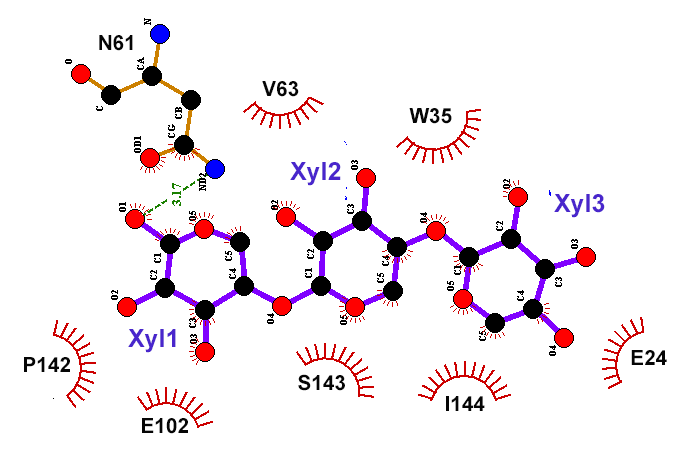


**(F)**

**(E)**

**Supplementary Fig. S3.** The three-dimensional HXYN2 model N°2 that was superimposed with the template GH11 xylanase SoXyn11B from *Streptomyces olivaceoviridis E-86* in complexes with alpha-L-3-arabinofuranosyl xylotetraose from [*https://www.rcsb.org*](https://www.rcsb.org) with identifiers (PDB: 7DFN) (**A**, **C**, **E**) and 4-o-methyl-alpha-D-3-glucuronopyranosyl xylotetraose (PDB: 7DFO) (**B**, **D**, **F**). Only the three-dimensional HXYN2 model and the molecules arabinofuranose (Abf1), xylo-oligosaccharide and xylose sugars 1, 2, 3, and 4 are shown. The surface of HXYN2 is represented in white and the strictly conserved residues of the aglycone and glycone regions are in red (**A** and **B**). Ligands are represented by the backbone in purple stick format. The xylo-oligosaccharide (Xyl1-Xyl2-Xyl3) and (Xyl1-Xyl2-Xyl3-Xyl4), with xylose sugars 3 (Xyl3) and 4 (Xyl4) in the reducing end sugar, are located in the glycone region. The structural arrangement of residues interacting with xylo-oligosaccharide in the glycone region is shown (**C** and **D**). Figures A-D were generated using *PyMol Molecular Graphics* *System* *version 2.1.1* ([*http://www.pymol.org*](http://www.pymol.org)). Residues are represented in lines and ligand in stick. Interactions between HXYN2 and xylo-oligosaccharide was represented by LIGPLOT^2^ (**E** and **F**). Residues and ligands are labelled; hydrogen bonds and hydrophobic interactions are represented as dashed lines and striped hemisphere, respectively.

***Determination of HXYN2 concentration***





**Supplementary Fig. S4.** Determination of HXYN2 concentration in the enzymatic reaction. Change in absorbance at 540 nm in a time of 0-180 seconds and concentration between 100 and 600 nM are shown.

***Analytical Ultracentrifugation of HXYN2***


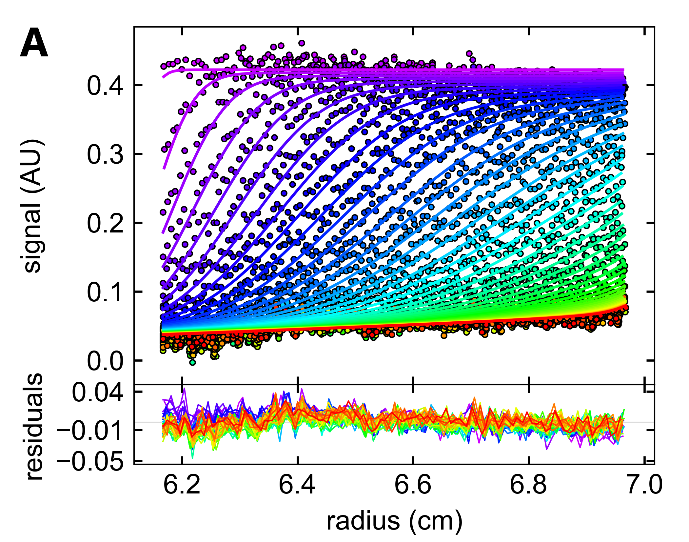

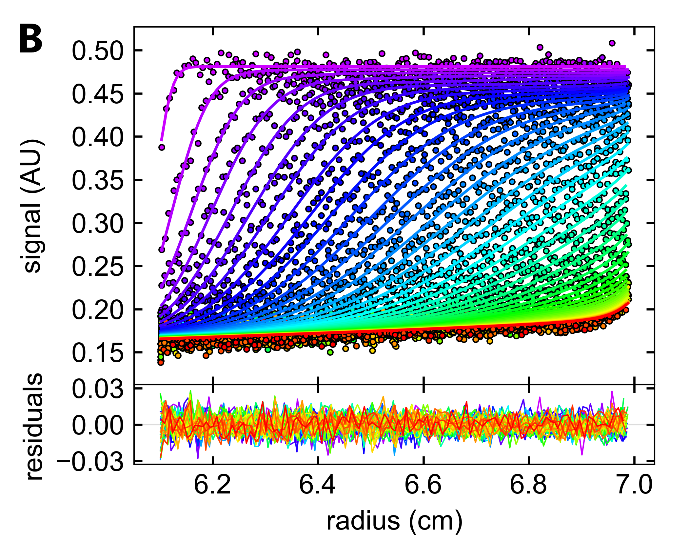

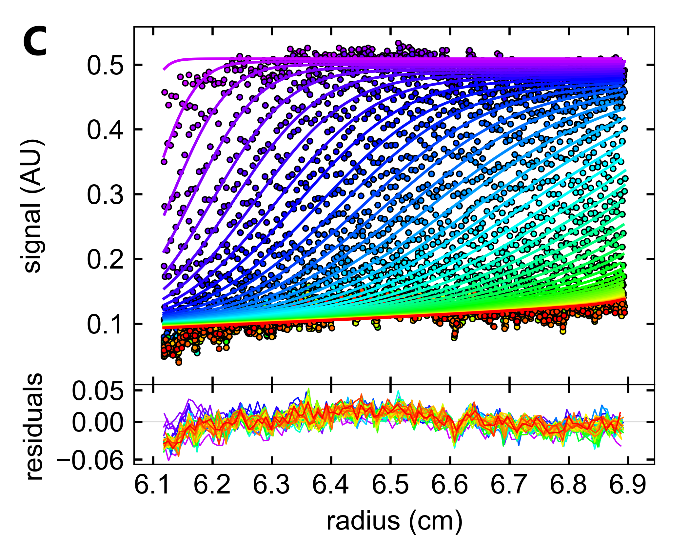

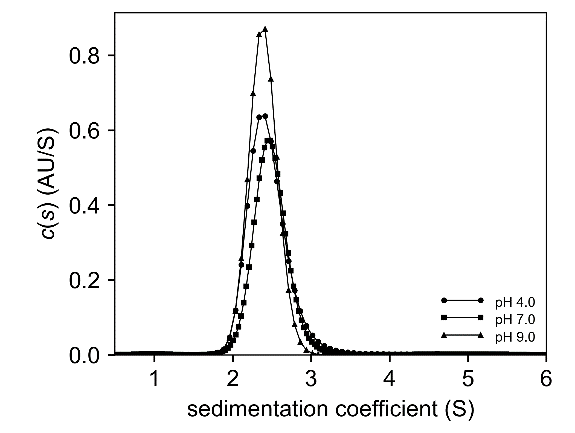


**D**

**Supplementary Fig. S5.** Analytical Ultracentrifugation of HXYN2 at acidic, neutral, and alkaline conditions. (**A**) pH 4.0, (**B**) pH 7.0 and (**C**) pH 9.0. Sedimentation profile of HXYN2 recorded by absorbance at 280 nm as a function of radial distance (radius) from the cell. At the bottom, the residual error values of the adjusted data are displayed. Coloured dots and lines represent collected and adjustment data at pH 4.0, 7.0, and 9.0, respectively. (**D**) Continuous distribution of HXYN2 sedimentation coefficient at pH 4.0, 7.0, and 9.0.

***Far-UV CD spectra of HXYN2***





| Secondary  Structure (%) | | pH  4.0 | | pH  6.0 | | pH  6.0_FA | | pH  7.0 | | pH  9.0 | |
| --- | --- | --- | --- | --- | --- | --- | --- | --- | --- | --- | --- |
| α-Helix | 2.6 | | 2.4 | | 2.1 | | 1.7 | | 0 | |  |
| β-Antiparallel | 51.3 | | 52.1 | | 42.5 | | 51.9 | | 53.4 | |  |
| β-Parallel | 0 | | 2.8 | | 0 | | 2.3 | | 0 | |  |
| Turn-β | 10.3 | | 9.3 | | 15.8 | | 8.8 | | 12.1 | |  |
| Random coil | 35.9 | | 33.4 | | 39.5 | | 35.3 | | 34.5 | |  |

**Supplementary Fig. S6.** Far-UV circular dichroism spectra of HXYN2 at different pHs, at 25 °C. HXYN2 (0.15 mg·mL^−1^) was solubilized in 5 mM sodium acetate (pH 4.0), Bis-Tris (pH 6.0), and Tris-HCl (pH 7.0 and 9.0). **Table inset:** Secondary structure content of HXYN2 at pH 4.0, 6.0, 7.0, and 9.0 and in the presence of FA at pH 6.0, estimated by Bestsel deconvolution program^3^.











**Supplementary Fig. S7.** Thermal denaturation of HXYN2 at pHs 4.0, 6.0, 7.0, and 9.0. (**A**–**D**) Far-UV CD spectra of HXYN2 as function of temperature ranging from 25 °C (solid line) to 95 °C (dashed line), at pH 4.0, 6.0, 7.0, and 9.0, respectively. The arrow indicates the decrease in molar ellipticity with increasing temperature.

***Molecular docking of HXYN2 with FA***


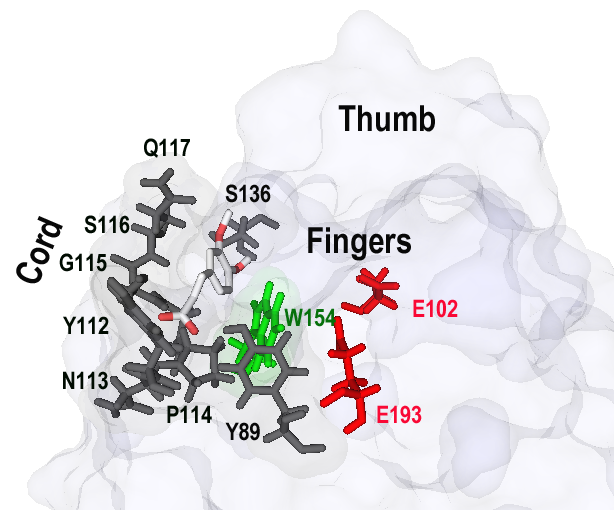

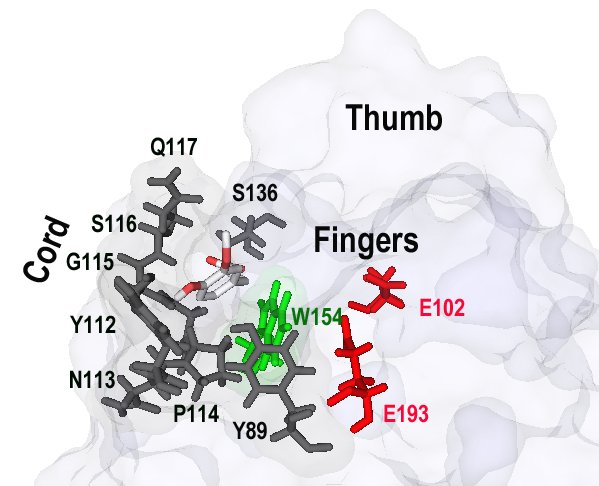


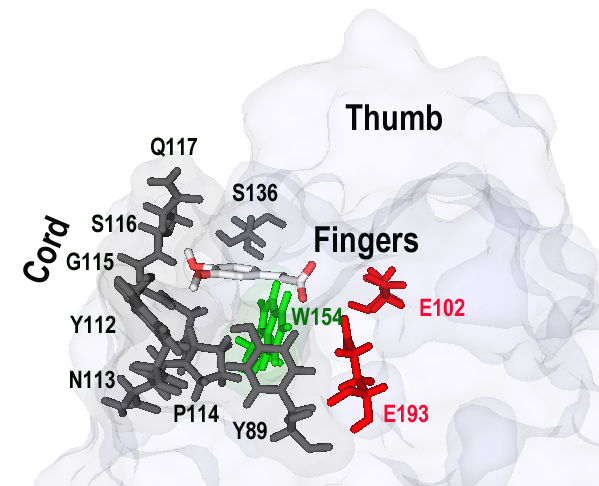

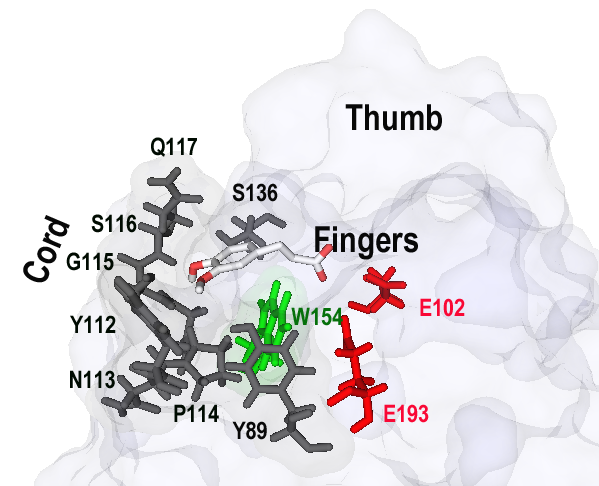


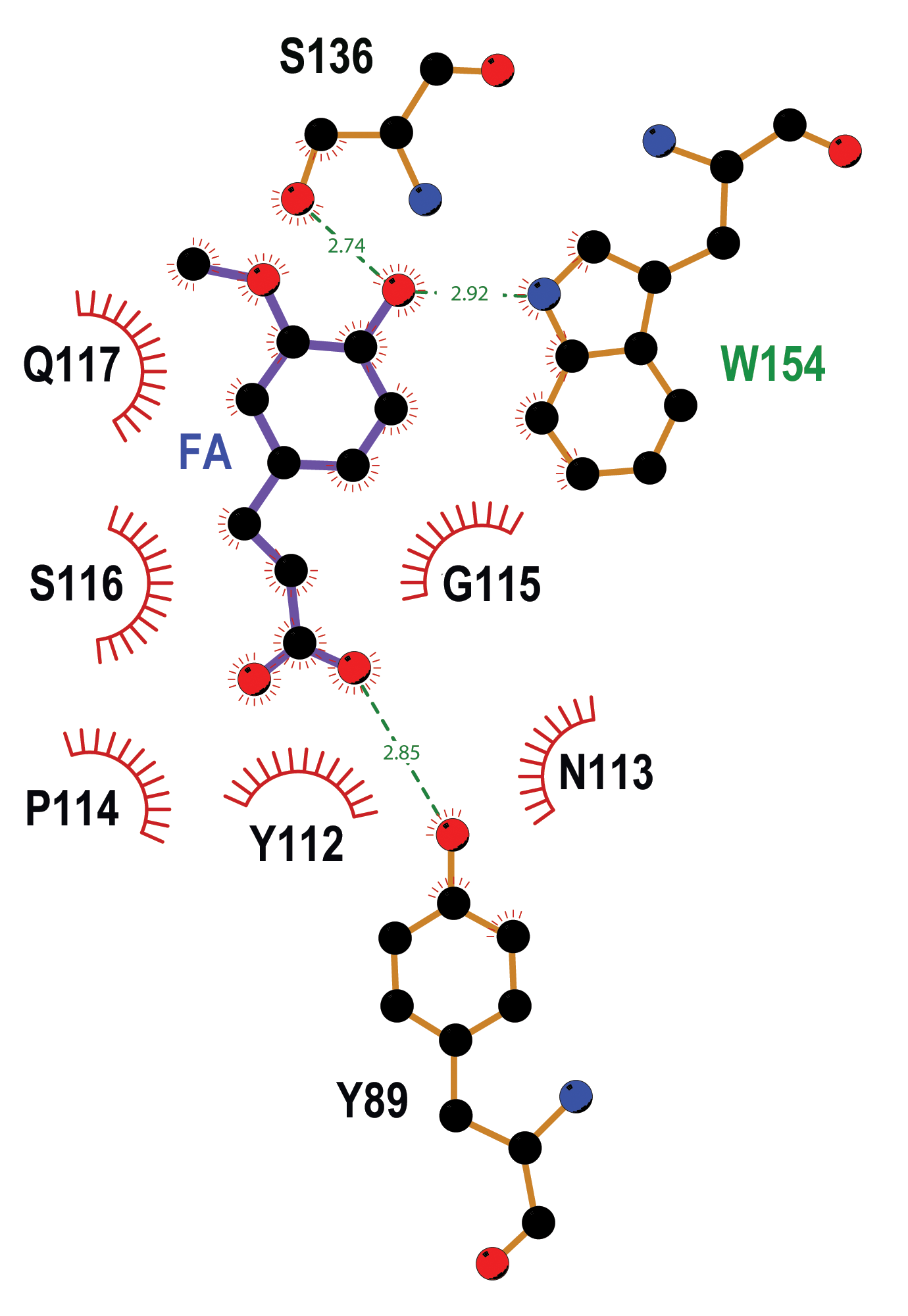

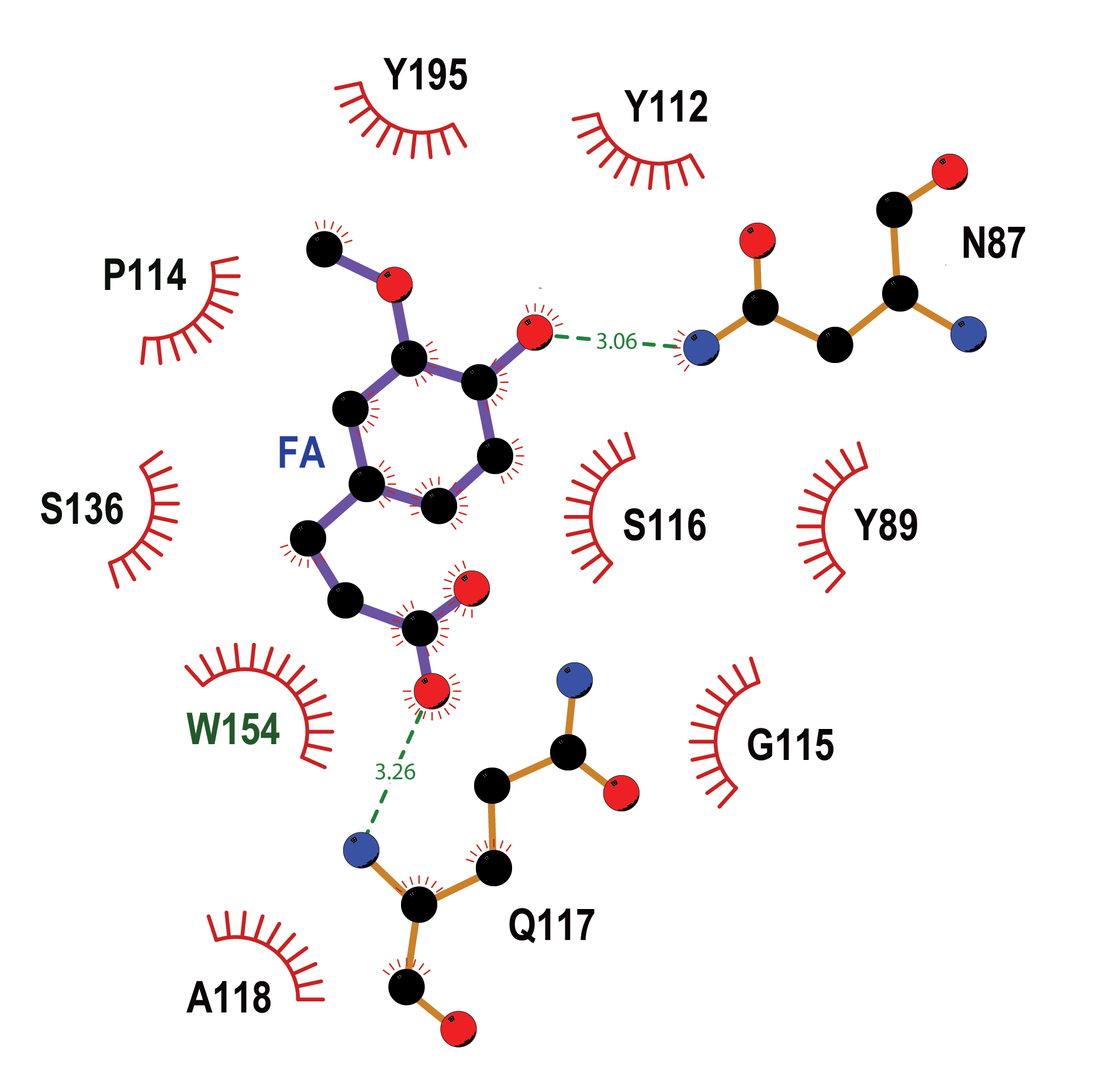

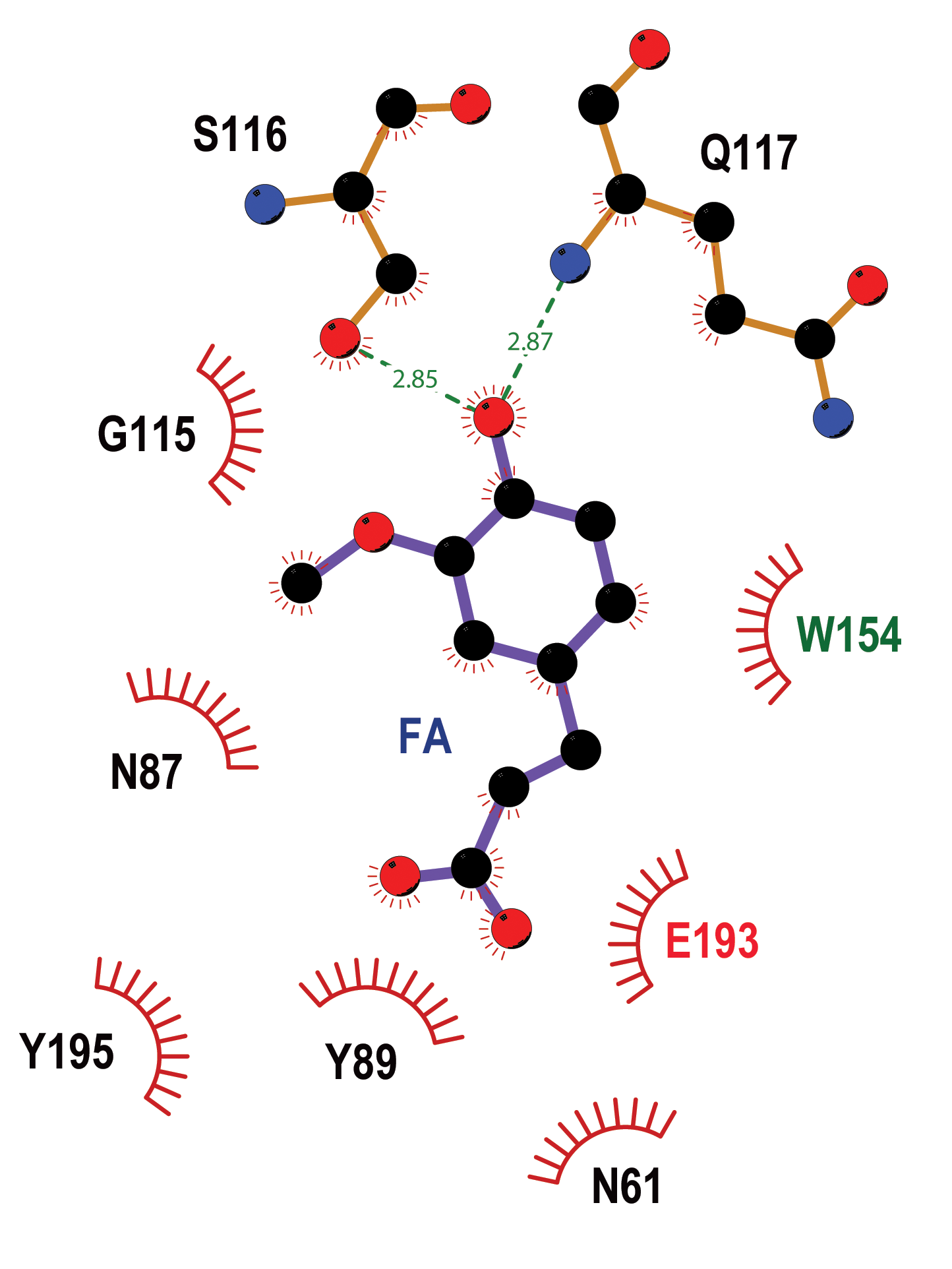

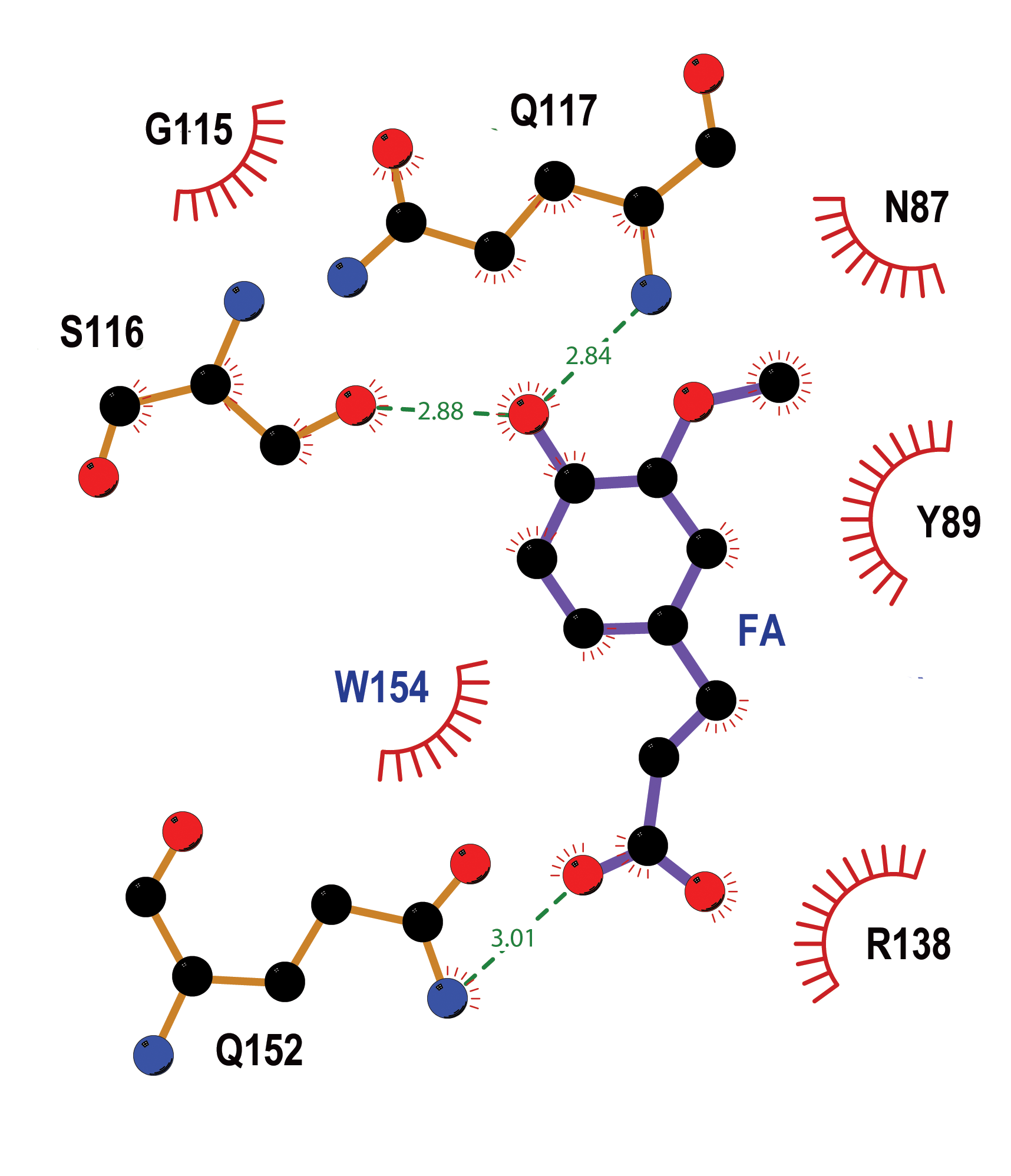


**(B)**

**(A)**

**(E)**

**(F)**

**(G)**

**(D)**

**(C)**

**(H)**

**Supplementary Fig S8.** Docking solutions of ferulic acid (FA) binding to the aglycone region of HXYN2. Panels A to D represent the relative position of FA in relation to the Thumb, Cord, and Fingers in the solutions 5, 7, 8, and 9, respectively. The surface of HXYN2 is represented in white, the catalytic Glu residues, interacting residues, and W154 are in red, gray, and green sticks, respectively. FA is represented with red and white stick for oxygen and carbon atoms, respectively. The interactions between HXYN2 and FA in solutions 5, 7, 8, and 9 are represented by LIGPLOT^2^ in panels E to H, respectively. FA and amino acid residues are labeled, and the hydrogen bonding and the hydrophobic interactions are represented as dashed lines and striped hemisphere, respectively.

| Purification steps | Volume  (mL) | Concentration  (mg/mL) | Total protein  (mg) | HXYN2*  (%) | HXYN2  (mg) | Activity  (U/mL) | Specific activity  (U/mg) | Yield  (%) | Purification factor |
| --- | --- | --- | --- | --- | --- | --- | --- | --- | --- |
| Crude extract | 700 | 0.3067 | 214.69 | 34.68 | 74.45 | 5516.3 | 17986.0 | 100 | 1 |
| Ultrafiltration | 15 | 3.4516 | 51.774 | 62.75 | 32.48 | 9247.1 | 2679.0 | 43.62 | 0.148 |
| Superdex 75 | 75 | 0.2950 | 22.13 | 100 | 22.13 | 8218.1 | 27857.97 | 29.72 | 1.54 |

* Percentage of HXYN2 estimated by densitometry (TotalLab version 1.10, Phoretix, USA) from SDS-PAGE gel stained with Coomassie brilliant blue.

**Supplementary Table S1.** Summary of parameters from purification steps of HXYN2

| Rank | Templ.  (PDB) | Enzyme | Family | Resl.  (Å) | Conf.  (%) | Cov.  (%) | Seq.id.  (%) | E-value  (x10^-73^) | Z-score |
| --- | --- | --- | --- | --- | --- | --- | --- | --- | --- |
| 1 | 3WP3_A | XylC | GH11 | 1.98 | 100.0 | 91.3 | 54.2 | 0.78 | 23.238 |
| 2 | 2VGD_A | NpXyn11A | GH11 | 1.8 | 100.0 | 93.3 | 52.8 | 3.7 | 23.038 |
| 3 | 2VUJ_A | EvXyn11 | GH11 | 1.8 | 100.0 | 92.8 | 52.8 | 3.0 | 23.028 |
| 4 | 5HXV_E | XylC | GH11 | 2.0 | 100.0 | 90.9 | 54.5 | 4.1 | 23.027 |
| 5 | 2DCJ_B | XynJ | GH11-XBM | 2.24 | 100.0 | 91.3 | 42.3 | 2.6 | 3.017 |

**Templ.**: Template pdb_ID from *https://www.rcsb.org/structure*, **Enzyme.**: Endo-1,4-β-xylanases, **family**: Glycoside hydrolase, **Resl.**: Resolution, **Conf.**: Confidence, **Cov.**: Coverage, **Seq.id.**: sequence identity **XBM**: *Xylan binding domain*

**Supplementary Table S2.** Structural templates automatically used to model the HXYN2 structure.

|  | | **Models** | **H-bond**  **(residues)** | **Hydrophobic Interactions**  **(residues)** | **Affinity**  **(kcal/mol)** | **rmsd l.b.***  **(Å)** | **rmsd u.b.****  **(Å)** |
| --- | --- | --- | --- | --- | --- | --- | --- |
| **Regions** | **I** | 1 | **P142** | S33, **W35**, V63, Y93, **E102**, Y104, **E193**, Y187, | -6.8 | 0 | 0 |
|  |  | 2 | Y93 | E24, **W35**, V63, **E102**, Y187 | -6.6 | 1.230 | 6.011 |
|  |  | 3 |  | **W35**, N61, V63, **P142**, **S143**, **E193** | -6.4 | 1.835 | 5.887 |
|  |  | 4 | Y187 | **W35**, N61, V63, Y93, **E102**, Y104, **P142**, **E193** | -6.0 | 1.943 | 2.407 |
|  |  | 5 | Y89, S136, **W154** | Y112, N113, P114, G115, S116, Q117 | -5.9 | 13.368 | 14.672 |
|  |  | 6 | S33, **W35**, Y93 | E24, **W34**, **P142**, **S143** | -5.7 | 3.473 | 6.324 |
|  |  | 7 | N87, Q117 | Y89, Y112, P114, G115, S116, A118, S136, Y195, **W154** | -5.7 | 12.873 | 14.222 |
|  |  | 8 | S116, Q117 | N61, N87, Y89, G115, **W154**, **E193**, Y195 | -5.3 | 9.844 | 12.319 |
|  |  | 9 | S116, Q117, Q152 | N87, Y89, G115, R138, **W154** | -5.1 | 8.473 | 12.046 |
|  | **II** | 1 | Q53, N83, E200 | Y81, E202 | -4.4 | 0 | 0 |
|  |  | 2 | N83, E200 | Y81, S201, D202 | -4.3 | 2.766 | 4.062 |
|  |  | 3 | N53, N83 | Y81, E200, S201, D202 | -4.3 | 1.726 | 5.505 |
|  |  | 4 | - | R55, **W56**, R57, N83, S198, G199, E200 | -4.2 | 7.794 | 8.969 |
|  |  | 5 | N53, N83 | Y81, E200, S201, D202 | -4.2 | 1.324 | 2.726 |
|  |  | 6 | N83 | N85, G199, E200 | -4.1 | 6.000 | 7.266 |
|  |  | 7 | - | R55, R57, N83, N85, S198, G199, E200 | -4.0 | 10.528 | 12.533 |
|  |  | 8 | T45, N53 | N18, N43, R55, E200 | -4.0 | 6.603 | 8.443 |
|  |  | 9 | N83 | Y81, D202 | -3.9 | 2.454 | 5.301 |
|  | **III** | 1 | S198 | R55, **W56**, R57, N58, N85, G199, E200 | -4.3 | 0 | 0 |
|  |  | 2 | S198, E200 | R55, **W56**, R57, N85, G199 | -4.2 | 1.534 | 2.197 |
|  |  | 3 | N18 | A16, N43, T45, N53, V54, R55, E200 | -4.1 | 10.378 | 13.571 |
|  |  | 4 | R57 | N41, N43, R55 | -4.0 | 6.508 | 8.090 |
|  |  | 5 | T45 | N18, N43, N53, V54, R55 | -3.8 | 11.268 | 13.320 |
|  |  | 6 | T45 | A16, N18, N43, N53, V54, R55 | -3.7 | 11.126 | 13.004 |
|  |  | 7 | N18, E200 | N43, T45, N53, V54, R55 | -3.6 | 10.498 | 11.221 |
|  |  | 8 | - | A16, N18, N43, T45, L47, R55, E200 | -3.6 | 10.300 | 13.399 |
|  |  | 9 | T45, N53, R55 | N43 | -3.4 | 10.523 | 12.057 |
|  | **IV** | 1 | N113, Y120 | P114, G115, S116, D132, F134 | -4.4 | 0 | 0 |
|  |  | 2 | N113, G115, A118 | S116, Y120, D132, F134 | -4.3 | 1.400 | 5.521 |
|  |  | 3 | G115, A118 | S116, Y120, D132, F134 | -4.2 | 2.964 | 4.377 |
|  |  | 4 | Y120 | D132, F134 | -4.2 | 4.293 | 5.340 |
|  |  | 5 | Y120, D132 | N119, F134 | -4.1 | 3.047 | 4.297 |
|  |  | 6 | Y120, D132 | A118, N119, F134 | -4.1 | 1.935 | 2.913 |
|  |  | 7 | Y120 | D132, F134 | -4.1 | 4.022 | 5.158 |
|  |  | 8 | Y120, K158 | T123, D132, F134 | -4.0 | 5.240 | 6.592 |
|  |  | 9 | A118 | G115, S116, Y120, D132, F134 | -4.0 | 2.757 | 5.123 |
|  | **V** | 1 | V6, N28 | F3, S11 | -5.4 | 0 | 0 |
|  |  | 2 | V6, N28, Y30, S50 | F3, D4, F5, S11 | -5.3 | 1.675 | 2.997 |
|  |  | 3 | V6, S50, Y204 | F3, G11, N28 | -5.2 | 1.803 | 2.524 |
|  |  | 4 | V6, Y30 | F3, F5, N28 | -4.9 | 1.640 | 2.957 |
|  |  | 5 | V6 | F3, F5, S11, N28 | -4.7 | 2.650 | 5.279 |
|  |  | 6 | - | F3, F5, V6, S11, N28 | -4.5 | 1.490 | 5.782 |
|  |  | 7 | V6 | F5, R8, S11, N28 | -4.5 | 3.819 | 6.091 |
|  |  | 8 | V6, Y204 | F3, F5, S11, N28 | -4.4 | 2.524 | 3.721 |
|  |  | 9 | N28 | F5, V6, P7, R8 | -4.2 | 3.834 | 5.873 |

*rmsd/lb (RMSD lower bound) and rmsd/ub (RMSD upper bound), differing in how the atoms are matched in the distance calculation.

**rmsd/ub matches each atom in one conformation with itself in the other conformation, ignoring any symmetry. rmsd' matches each atom in one conformation with the closest atom of the same element type in the other conformation (rmsd' cannot be used directly, because it is not symmetric). rmsd/lb is defined as follows: rmsd/lb(c1, c2) = max(rmsd'(c1, c2), rmsd'(c2, c1)). If the ligand has no internal symmetry, rmsd/ub is the value to use. If the ligand has internal symmetry, this would give different values depending on the ligand is rotates, therefore rmsd/lb gives a better ranking of docking solutions. *http://mgldev.scripps.edu/pipermail/autodock/2009-March/005265.html*

**Supplementary Table S3.** Interaction parameters of HXYN2 in complex with FA estimated by molecular docking.

**References**

1. Lowry, O. H., Rosebrough, N. J., Farr, A. L. & Randall, R. J. Protein measurement with the Folin phenol reagent. *J Biol Chem*. **193**, 265-275 (1951).

2. Wallace, A. C., Laskowski, R. A. & Thornton, J. M. LIGPLOT: a program to generate schematic diagrams of protein-ligand interactions. *Protein Eng*. **8**, 127-134 (1995).

3. Micsonai, A.*, et al.* Accurate secondary structure prediction and fold recognition for circular dichroism spectroscopy. *Proc Natl Acad Sci U S A*. **112**, E3095-3103 (2015).
